# High-Content Screening in Zebrafish Embryos Identifies Butafenacil as a Potent Inducer of Anemia

**DOI:** 10.1371/journal.pone.0104190

**Published:** 2014-08-04

**Authors:** Jessica K. Leet, Casey D. Lindberg, Luke A. Bassett, Gregory M. Isales, Krystle L. Yozzo, Tara D. Raftery, David C. Volz

**Affiliations:** Department of Environmental Health Sciences, Arnold School of Public Health, University of South Carolina, Columbia, South Carolina, United States of America; National University of Singapore, Singapore

## Abstract

Using transgenic zebrafish (*fli1:egfp*) that stably express enhanced green fluorescent protein (eGFP) within vascular endothelial cells, we recently developed and optimized a 384-well high-content screening (HCS) assay that enables us to screen and identify chemicals affecting cardiovascular development and function at non-teratogenic concentrations. Within this assay, automated image acquisition procedures and custom image analysis protocols are used to quantify body length, heart rate, circulation, pericardial area, and intersegmental vessel area within individual live embryos exposed from 5 to 72 hours post-fertilization. After ranking developmental toxicity data generated from the U.S. Environmental Protection Agency's (EPA's) zebrafish teratogenesis assay, we screened 26 of the most acutely toxic chemicals within EPA's ToxCast Phase-I library in concentration-response format (0.05–50 µM) using this HCS assay. Based on this screen, we identified butafenacil as a potent inducer of anemia, as exposure from 0.39 to 3.125 µM butafenacil completely abolished arterial circulation in the absence of effects on all other endpoints evaluated. Butafenacil is an herbicide that inhibits protoporphyrinogen oxidase (PPO) – an enzyme necessary for heme production in vertebrates. Using *o*-dianisidine staining, we then revealed that severe butafenacil-induced anemia in zebrafish was due to a complete loss of hemoglobin following exposure during early development. Therefore, six additional PPO inhibitors within the ToxCast Phase-I library were screened to determine whether anemia represents a common adverse outcome for these herbicides. Embryonic exposure to only one of these PPO inhibitors – flumioxazin – resulted in a similar phenotype as butafenacil, albeit not as severe as butafenacil. Overall, this study highlights the potential utility of this assay for (1) screening chemicals for cardiovascular toxicity and (2) prioritizing chemicals for future hypothesis-driven and mechanism-focused investigations within zebrafish and mammalian models.

## Introduction

Baseline toxicity and chemical mode-of-action (MOA) data are lacking for the majority of chemicals currently in commerce within the United States and around the world. In most cases, this uncertainty reflects the fact that conventional, animal-based toxicity tests are resource-intensive, require high animal numbers, and, due to a principal focus on apical endpoints, provide little to no information about chemical MOA. Based on these limitations, increased public concern, and an interest in reducing animal usage, organizations around the world have advocated for the development and application of rapid and cost-effective assays that support MOA- or pathway-based chemical screening and prioritization for further hazard characterization [1], [2], [3].

Zebrafish are well studied and established as a model species for developmental and environmental toxicology, and have become a desirable alternative to mammalian animal models for toxicity testing – especially since embryos and eleutheroembryos are considered non-protected life stages within the European Union and United States [4], [5], . Due to transparency, small size, and rapid development *ex utero*, zebrafish are highly amenable to whole-organism imaging during embryogenesis and, as a result, high-content screening (HCS) assays for identifying and prioritizing hazardous compounds for further testing. To this end, using transgenic zebrafish (*fli1:egfp*) that stably express enhanced green fluorescent protein (eGFP) within vascular endothelial cells, we recently developed and optimized a 384-well HCS assay that enables us to screen and identify chemicals affecting cardiovascular development and function at non-teratogenic concentrations [8]. Within this assay, automated image acquisition procedures and custom image analysis protocols are used to quantify body length, heart rate, circulation, pericardial area, and intersegmental vessel area within individual live embryos exposed from 5 to 72 hours post-fertilization (hpf). Although other HCS zebrafish assays have been developed for evaluating cardiac function or cardiovascular morphology [9], [10], [11], our assay incorporates both functional and morphological endpoints, provides increased initial sample sizes (32 embryos per treatment), and allows us to simultaneously test a wide range of concentrations (12 treatment groups, including one vehicle control).

The objective of this study was to evaluate the potential utility of this HCS assay for screening and prioritizing chemicals for cardiovascular toxicity testing. As this was a pilot study, we restricted our screen to the most acutely toxic chemicals from the U.S. Environmental Protection Agency's (EPA's) ToxCast Phase-I chemical library in order to minimize potential false negative results due to limited chemical transfer across the embryonic chorion. Therefore, chemicals within the EPA's ToxCast Phase-I chemical library were ranked from most to least acutely toxic based on available data for zebrafish survival and gross malformations present at 6 days post fertilization (dpf) following an 8-hpf to 5-dpf exposure [12]. Using protocols described in Yozzo et al. [8], 26 of the most acutely toxic chemicals within this library (Table S1) were first screened in concentration-response format (0.05–50 µM) to evaluate the ability of this assay to detect cardiovascular toxicity at non-teratogenic concentrations. Based on results from this pilot screen, we then conducted initial hit validation studies to (1) determine whether exposure to chemicals with a common site of action resulted in similar effects on cardiovascular development and (2) begin characterizing the potential mechanism leading to cardiovascular toxicity.

## Materials and Methods

### Animals

For all assays described below, we relied on a robust line of transgenic zebrafish (*fli1:egfp*) that stably express eGFP within vascular endothelial cells [13]. Adult *fli1:egfp* zebrafish were maintained on a 14-h∶10-h light∶dark cycle within a zebrafish stand-alone system (Aquatic Habitats, Inc., Apopka, FL) containing photoperiod light-cycle enclosures and recirculating conditioned reverse osmosis (RO) water (∼27–28°C). Adult females and males were bred directly on-system using in-tank breeding traps suspended within 3-L tanks, directly within a Mini Mass Embryo Production System (Aquatic Habitats), or bred off-system within a light- and temperature-controlled incubator using breeding traps suspended within 1-L tanks. For all experiments described below, newly fertilized eggs were staged according to previously described methods [14]. All fish were handled and treated in accordance with approved Institutional Animal Care and Use Committee protocols at the University of South Carolina – Columbia (protocol # 2021-100372-072511). To minimize discomfort and distress, zebrafish husbandry and euthanasia methods were consistent with the National Research Council's *2011 Guide for the Care and Use of Laboratory Animals* and American Veterinary Medical Association's *2007 Guidelines on Euthanasia*, respectively.

### Chemicals

Chemicals were purchased from ChemService, Inc. (West Chester, PA) and Sigma Aldrich (St. Louis, MO) (Table S1). Stock solutions of each chemical were prepared by dissolving chemicals in high performance liquid chromatography (HPLC)-grade dimethyl sulfoxide (DMSO) (50 mM), and then performing two-fold serial dilutions into DMSO to create stock solutions for each working solution. All stock solutions were stored at room temperature within 2-mL amber glass vials containing polytetrafluoroethylene-lined caps. For each individual plate, working solutions of tricaine methanesulfonate (MS-222) (Western Chemical, Inc., Ferndale, WA) were freshly prepared by dissolving MS-222 into embryo media (EM) (5 mM NaCl, 0.17 mM KCl, 0.33 mM CaCl_2_, 0.33 mM MgSO_4_), and working solutions of all treatments were freshly prepared by spiking stock solutions into EM, resulting in 0.1% DMSO within all vehicle control and treatment groups.

### High-content screening (HCS) assay

#### Exposure setup

Black 384-well microplates containing 0.17-mm glass-bottom wells (Matrical Bioscience, Spokane, WA) were used for all HCS assays. Immediately following spawning, newly fertilized eggs were collected and placed in groups of approximately 50 per glass petri dish within a light- and temperature-controlled incubator until 5 hpf. For each HCS assay, 384 viable *fli1:egfp* embryos were arrayed at 5 hpf into a 384-well plate (one embryo per well) containing 50 µL per well of vehicle (0.1% DMSO) or treatment solution, and then incubated at 28°C under a 14-h∶10-h light∶dark cycle and static conditions until 72 hpf.

#### Image acquisition

At 72 hpf, the plate was removed from the incubator, and zebrafish embryos were anesthetized with 100 mg/L MS-222 by adding 25 µL of 300 mg/L MS-222 to 50 µL of vehicle or treatment solution. The plate was then centrifuged at 200 rpm for 2 minutes to help orient hatched embryos into right or left lateral recumbency. Using automated image acquisition protocols and parameters previously optimized [8] for our ImageXpress Micro (IXM) Widefield High-Content Screening System (Molecular Devices, Sunnyvale, CA), each embryo was imaged to analyze the following endpoints: heart rate, arterial circulation, pericardial area, body length, and intersegmental vessel area. During the entire image acquisition period, internal temperature within the IXM system was maintained between 25–27°C by removing panels on both sides of the IXM system and blowing air from left to right through the IXM with a portable fan; internal temperature was monitored and recorded at initiation and termination of each imaging protocol using a digital thermometer. In accordance with National Institutes of Health (NIH) guidelines [15], 72-hpf embryos were then euthanized by placing the plate at 4°C for 30 minutes.

#### Data extraction

Within MetaXpress 4.0.0.24 software (Molecular Devices, Sunnyvale, CA), custom journal scripts for extraction of heart rate, arterial circulation, pericardial area, body length, and intersegmental vessel area data were used as previously described [8]. Prior to data extraction, stream acquisitions within each well were inspected within MetaXpress to assess embryo orientation and survival. Coagulated embryos, unhatched embryos, grossly malformed embryos, or developed embryos lacking a heartbeat were considered dead. Using these survival criteria, only hatched and live embryos positioned in right or left lateral recumbency were analyzed. Interactive semi-automated journal scripts were used to isolate regions of interest and quantify heart rate, arterial circulation, pericardial area, and intersegmental vessel area, whereas a fully automated journal script was used to quantify body length. Examples of raw and analyzed images for each endpoint as well as additional details of the data extraction and analysis process are described within Yozzo et al. [8].

### Hemoglobin staining

Based on protocols previously described by Paffett-Lugassy and Zon [16], *o*-dianisidine staining was used to detect the presence of hemoglobin within intact 30-, 48-, and 72-hpf zebrafish embryos exposed to vehicle (0.1% DMSO) or butafenacil (0.195 and 0.78 µM). Dechorionated or hatched embryos were stained in the dark for 30 min at room temperature within a solution containing *o*-dianisidine (0.6 mg/ml), 0.01 M sodium acetate (pH 4.5), 0.65% H_2_O_2_, and 40% (vol/vol) ethanol. Once stained, embryos were washed with RO water and then fixed in 4% paraformaldehyde for at least 1 h at room temperature. Pigments were removed from fixed embryos by incubating in a solution of 0.8% KOH, 0.9% H_2_O_2_, and 0.1% Tween-20 for 30 min at room temperature. Embryos were then washed with phosphate-buffered saline (PBS) containing 0.1% Tween-20, and then fixed again in 4% paraformaldehyde for at least 3 h before storage in PBS at 4°C. All embryos were oriented in dorsal recumbency and imaged using an Olympus BX51 Research System Microscope equipped with an Olympus DP72 Digital Color Camera and Olympus DP2-BSW Imaging Software (Olympus Corp., Center Valley, PA). Images were analyzed using a color range tool within Adobe Photoshop CS4 (San Jose, CA). Sample colors were selected within vehicle control images to represent the *o*-dianisidine stain, and then control color samples were used to measure the stained area on the ventral side of the yolk sac and pericardial region of embryos across all treatments.

### Statistical analyses

All statistical procedures were performed using SPSS Statistics 19.0 (Chicago, IL). A general linear model (GLM) analysis of variance (ANOVA) (α = 0.05) was used for all data, as these data did not meet the equal variance assumption for non-GLM ANOVAs. Pair-wise Tukey-based multiple comparisons of least square means were performed to identify significant treatment-related effects. To estimate the median lethal concentration (LC_50_) after static exposure to each chemical from 5 to 72 hpf, a four-parameter concentration-response curve was fit to percent mortality data using log-transformed chemical concentrations within Prism 6.0 (GraphPad Software Inc., La Jolla, CA).

## Results

### Chemical screening

**Figure 1:**
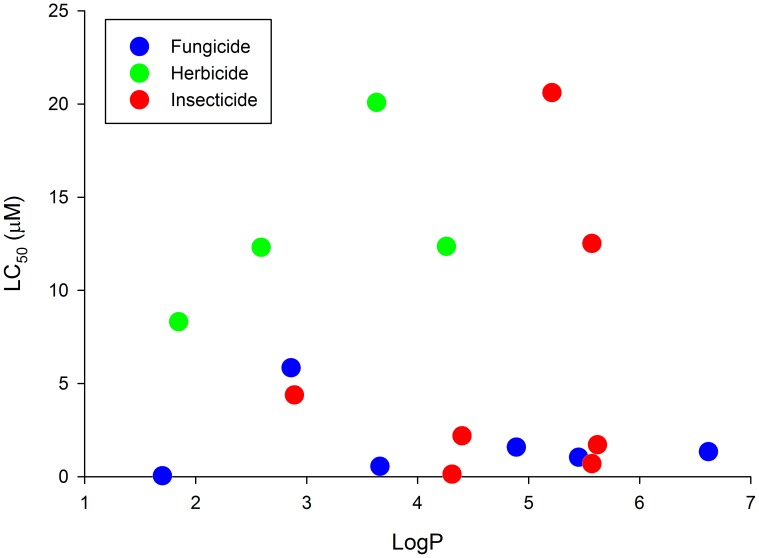
Lethal concentration (LC_50_) vs. octanol∶water coefficient (LogP) for 17 chemicals screened from the EPA's ToxCast Phase-I library.

**Figure 2:**
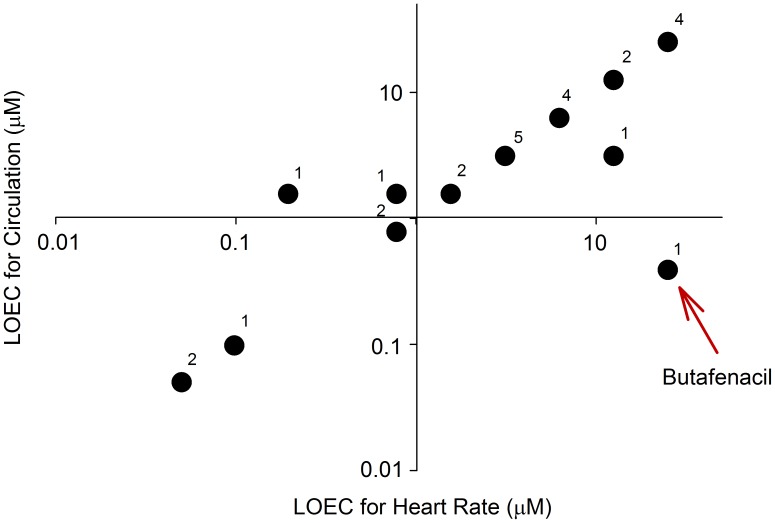
Comparison of lowest observable effect concentrations (LOECs) for heart rate and circulation for 26 of the most acutely toxic chemicals from the EPA's ToxCast Phase-I library. Butafenacil was the only chemical with a concentration-dependent effect on circulation in the absence of effects on heart rate. Numbers above the points denote the number of chemicals each point represents.

For this study, we first identified 31 of the most acutely toxic chemicals from the EPA's ToxCast Phase-I chemical library (Table S1). Two chemicals (pyridaben and cypermethrin) were previously screened using this HCS assay [8]. In addition, two chemicals (tefluthrin and milbemectin) were not commercially available (as of August 2013), and fentin was insoluble in DMSO at concentrations as low as 2.5 mM. Therefore, these five chemicals were not included in the final pilot screen for this study, resulting in a total of 26 chemicals screened in concentration-response format. Microsoft Excel spreadsheets containing raw data for all HCS assays are provided within Dataset S1. To determine targeted effects on the cardiovascular system, heart rate, arterial circulation, pericardial area, and intersegmental vessel area were quantified within treatment groups with >70% survival and/or <10% decrease in body length relative to vehicle controls [8]. All chemicals in this initial screen had at least one concentration that resulted in an effect on either body length or survival (0.05–50 µM) (Figures S1, S2, S3, S4, S5, S6, S7, S8, S9, S10, S11, S12, S13, S14, S15, S16, S17, S18, S19, S20, S21, S22, S23, S24, S25, S26). Of the chemicals tested, thiram was the most acutely toxic based on survival, as exposure to greater than 0.02 µM – the highest concentration analyzed – resulted in <70% survival (Figure S2). Based on survival data, we calculated an LC_50_ for approximately 65% of the chemicals screened (17 chemicals) and, for these chemicals, found no relationship between the chemical-specific LC_50_ and octanol∶water partition coefficient (logP) (Figure 1). We then compared the lowest observable effect concentrations (LOECs) for functional endpoints (heart rate and circulation) to identify the ability of this assay to discriminate chemicals with specific effects on cardiac vs. vascular function. Based on this comparison, butafenacil was identified as the only chemical that significantly impacted circulation in the absence of effects on heart rate (Figure 2).

**Figure 3:**
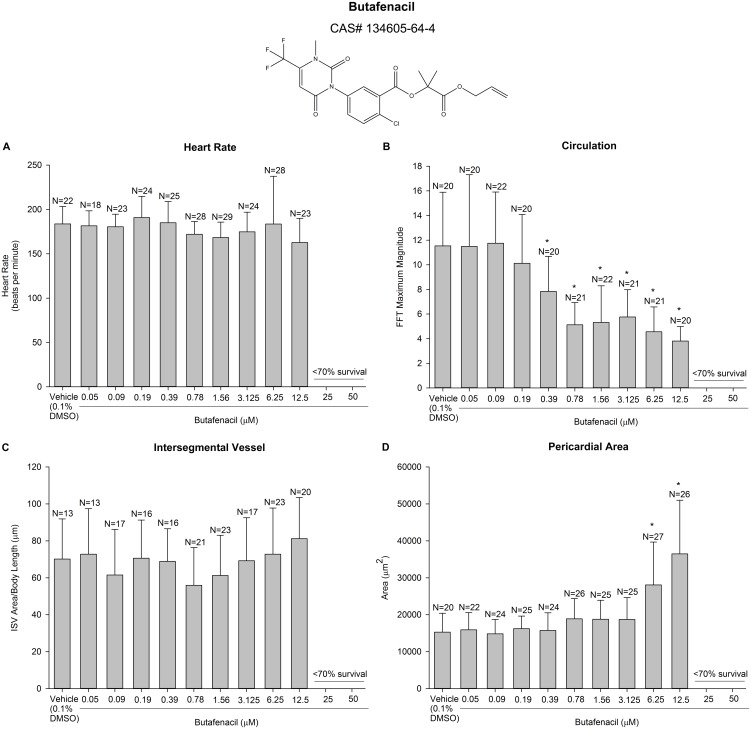
Butafenacil exposure resulted in a concentration-dependent effect on circulation. Based on decision criteria defined by Yozzo et al. [8], concentrations with <70% survival were not analyzed for potential effects on circulation, pericardial area, heart rate, or intersegmental vessel area. An asterisk denotes a significant difference from vehicle controls (p<0.05). N = final number of embryos analyzed per treatment.

Butafenacil is an herbicide that inhibits protoporphyrinogen oxidase (PPO) [20] – an enzyme necessary for heme production in vertebrates – and was the only chemical to induce a significant concentration-dependent effect on any of the endpoints measured within our HCS assay. Survival was <70% for butafenacil concentrations higher than 12.5 µM (Figures S3). Therefore, 12.5 µM was the highest concentration analyzed for all endpoints. At 0.39 µM and higher, butafenacil exposure resulted in a concentration-dependent decrease in arterial circulation in the absence of effects on heart rate and intersegmental vessel area (Figure 3). Although butafenacil exposure increased pericardial area, this effect was only significant at the two highest concentrations analyzed (6.25 and 12.5 µM). As indicated by our quantitative data, exposure of embryos to butafenacil at 0.39 µM and higher resulted in visibly decreased circulation (Video S1) compared to embryos exposed to vehicle alone (Video S2).

To determine whether other commercially available PPO inhibitors mimicked butafenacil-induced anemia, we relied on classifications defined by the Herbicide Resistance Action Committee (http://www.hracglobal.com/Education/ClassificationofHerbicideSiteofAction.aspx), Insecticide Resistance Action Committee (http://www.irac-online.org/documents/moa-classification/?ext=pdf), and Fungicide Resistance Action Committee (http://www.frac.info/publication/anhang/FRAC%20Code%20List%202013-update%20April-2013.pdf) to first assign MOAs to all pesticides within the ToxCast Phase-I chemical library and, if necessary, then test additional PPO inhibitors not included within our original pilot screen. In addition to butafenacil, five PPO inhibitors (fluthiacet-methyl, pyraflufen-ethyl, lactofen, flufenpyr-ethyl, and carfentrazone-ethyl) were included within our initial pilot screen; six additional (but less acutely toxic) PPO inhibitors (flumiclorac-pentyl, oxadiazon, flumioxazin, oxyfluorfen, sulfentrazone, and acifluorfen) not included within our initial pilot screen were also tested using our HCS assay (Figures S27, S28, S29, S30, S31, S32). Out of 11 PPO inhibitors screened (excluding butafenacil), flumioxazin was the only chemical to induce anemia and significantly decrease arterial circulation at more than three concentrations (Figure S29), albeit the phenotype was not as severe compared to butafenacil (Figure 3 and Figure S3).

### Hemoglobin staining

**Figure 4:**
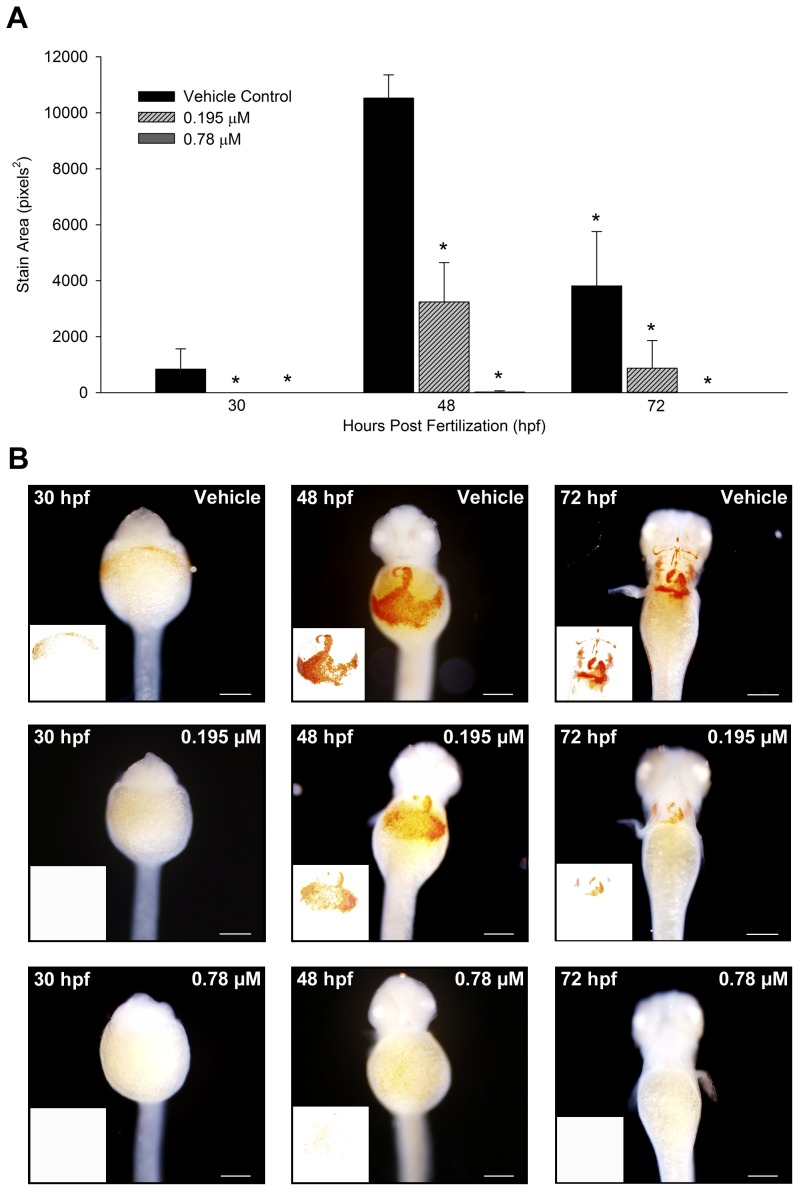
*o*-Dianisidine staining in embryos exposed to vehicle (0.1% DMSO) or butafenacil (A), and representative images at each time point for vehicle, 0.195, and 0.78 µM butafenacil (B). All embryos were oriented in dorsal recumbency for imaging. An asterisk denotes a significant difference from vehicle controls (p<0.05). White boxes represent stained areas within representative images. Scale bar = 200 µm.

The presence of hemoglobin was assessed in embryos exposed to vehicle (0.1% DMSO), 0.195, or 0.78 µM butafenacil from 5 hpf to either 30, 48, or 72 hpf. Hemoglobin-positive cells appeared within vehicle-exposed embryos shortly after circulation commenced at 30 hpf, but these cells were absent following exposure to 0.195 or 0.78 µM butafenacil (Figure 4). At 48 and 72 hpf, embryos exposed to 0.195 µM butafenacil resulted in significantly decreased hemoglobin compared to vehicle controls (Figure 4), whereas embryos exposed to 0.78 µM butafenacil showed negligible or no *o*-dianisidine staining, indicating complete abolishment of hemoglobin due to butafenacil exposure (Figure 4).

## Discussion

In this study, we leveraged our recently developed and optimized 384-well HCS assay to screen and identify chemicals affecting cardiovascular development and function at non-teratogenic concentrations. After ranking acute toxicity data generated from the U.S. EPA's zebrafish teratogenesis assay, we first screened 26 of the most acutely toxic chemicals within EPA's ToxCast Phase-I chemical library in concentration-response format (0.05–50 µM). Based on this screen, we identified butafenacil as a potent inducer of anemia, as exposure to 0.39–3.125 µM butafenacil completely abolished arterial circulation in the absence of effects on all other endpoints evaluated. Using *o*-dianisidine staining, we then revealed that severe butafenacil-induced anemia in zebrafish was due to a complete loss of hemoglobin following exposure during early development.

All ToxCast Phase-I chemicals in our initial pilot screen resulted in significant effects on body length or survival at 50 µM or lower. Therefore, an absence of targeted effects on the cardiovascular system was not likely due chemical sorption to well walls or limited uptake across the embryonic chorion. Although previous research has suggested that acute toxicity (LC_50_) and hydrophobicity (logP) are positively correlated [12], findings from this study and others [17], [18] suggest that there is no relationship between these two parameters (Figure 1). However, these discrepancies may be due to differences in experimental design such as the use of glass inserts within 96-well plates vs. adding treatment solutions directly to wells of 96- and 384-well plates. Interestingly, we were unable to determine an LC_50_ for six chemicals screened, five of which have a logP>5.5 and one of which has a logP<1, suggesting that there may be an optimal logP range for chemicals tested within our assay.

Based on our screen, butafenacil was the only chemical to induce a significant concentration-dependent effect on any of the endpoints evaluated. Butafenacil is an herbicide that inhibits PPO, an enzyme that catalyzes oxidation of protoporphyrinogen IX to protoprophyrin IX – the penultimate step of chlorophyll and heme biosynthesis in plants and animals, respectively [19]. As a result, this post-emergent herbicide is phytotoxic on contact and provides rapid knockdown of broadleaf and grass weeds [20]. While there are currently no registered butafenacil-containing products within the United States, butafenacil is registered for use within Australia, Argentina, Brazil, Japan, Switzerland, and Thailand [20]. Registered uses include cotton and citrus, as well as uses on cereal grains (except rice) up to 100 to 200 g ai/ha [20].

Given that butafenacil is a PPO inhibitor and completely eliminated hemoglobin production and circulation, butafenacil likely blocked heme biosynthesis and, as a result, hemoglobin and red blood cell production within zebrafish embryos. Similarly, butafenacil exposure in rodent models (rats and mice) resulted in decreased hemoglobin, hematocrit, and mean corpuscular volume [21]. While ruptured vessels and extravasation of blood cells may also lead to abolishment of circulation, butafenacil-induced anemia was likely not mediated by this MOA, as hemorrhaging was absent based on images generated from hemoglobin staining and our HCS assay. Although circulation was abolished in embryos exposed to 0.39 to 3.125 µM butafenacil, these embryos developed in the absence of gross malformations – a finding that is consistent with zebrafish mutants that develop little to no blood cells. These mutants appear normal for the first five days during development, but fail to inflate a swim bladder and die within two weeks [22]. The lack of developmental malformations is because oxygen demands of developing zebrafish are met by bulk diffusion until about two weeks post-fertilization [23]. By 12 to 14 dpf, functional hemoglobin is needed for proper swim bladder inflation and the transition to gill uptake and convective oxygen transport [24]; therefore, these zebrafish mutants do not survive beyond approximately 14 dpf. Similarly, if irreversible, butafenacil-exposed embryos would likely not survive after two weeks following the transition from passive oxygen diffusion to convective oxygen transport.

Out of 11 PPO inhibitors screened (excluding butafenacil), flumioxazin was the only PPO inhibitor to induce an anemic phenotype similar to butafenacil, albeit the flumioxazin-induced phenotype was not as severe as butafenacil. With the possible exception of sulfentrazone and acifluorfen (the least acutely toxic chemicals), it is unlikely that differential uptake across the chorion accounts for an absence of anemia within embryos exposed to the remaining eight PPO inhibitors screened, as significant effects on one or more endpoints were observed within at least one concentration tested within our assay. Rather, potency differences may be attributable to relative differences in chemical affinity to zebrafish PPO, but this was not directly tested within our study or, to date, within any other laboratories. While there are over 700 assays within the ToxCast program, to our knowledge none of these assays directly address chemical-mediated PPO inhibition nor impacts on hemoglobin production, suggesting that our targeted, whole-organism HCS assay has the potential to identify a unique mechanism (PPO inhibition) and phenotype (anemia) not currently covered by the large battery of existing ToxCast assays.

In summary, results from this study suggest that our HCS assay not only has potential for screening potential chemicals for cardiovascular toxicity, but also has potential for identifying chemicals impacting hematopoiesis. Adverse outcomes due to inhibition of PPO are detectable by this assay, and PPO is functionally conserved between zebrafish and humans. Moreover, PPO is established as an important target for certain human blood disorders, such as variegate porphyria for which preventative therapies are still needed [25]. While certain zebrafish mutant strains with disrupted blood cell development or differentiation are available, these strains have mainly been used to investigate the molecular mechanisms of hematopoiesis rather than HCS applications [26], [27]. Therefore, using *fli1:egfp* zebrafish and butafenacil as a positive control, this HCS assay may provide a robust whole-organism-based platform for pre-clinical toxicology and/or drug discovery efforts focused on rapid identification of pro- or anti-anemic chemicals.

## Supporting Information

Figure S1 **Rotenone did not have a concentration dependent effect on any endpoints.** Based on decision criteria defined by Yozzo et al. [8], hashed bars represent concentrations that were not analyzed for potential effects on circulation, pericardial area, heart rate, or intersegmental vessel area. An asterisk denotes a significant difference from vehicle controls (p<0.05). N = final number of embryos analyzed per treatment.
(TIF)

Figure S2 **Thiram did not have a concentration dependent effect on any endpoints.** Based on decision criteria defined by Yozzo et al. [8], hashed bars represent concentrations that were not analyzed for potential effects on circulation, pericardial area, heart rate, or intersegmental vessel area. An asterisk denotes a significant difference from vehicle controls (p<0.05). N = final number of embryos analyzed per treatment.
(TIF)

Figure S3 **Butafenacil significantly decreased circulation without having a concentration dependent effect on any other endpoint.** Based on decision criteria defined by Yozzo et al. [8], hashed bars represent concentrations that were not analyzed for potential effects on circulation, pericardial area, heart rate, or intersegmental vessel area. An asterisk denotes a significant difference from vehicle controls (p<0.05). N = final number of embryos analyzed per treatment.
(TIF)

Figure S4 **Flumetralin did not have a concentration dependent effect on any endpoints.** Based on decision criteria defined by Yozzo et al. [8], hashed bars represent concentrations that were not analyzed for potential effects on circulation, pericardial area, heart rate, or intersegmental vessel area. An asterisk denotes a significant difference from vehicle controls (p<0.05). N = final number of embryos analyzed per treatment.
(TIF)

Figure S5 **Fluthiacet-methyl did not have a concentration dependent effect on any endpoints.** Based on decision criteria defined by Yozzo et al. [8], hashed bars represent concentrations that were not analyzed for potential effects on circulation, pericardial area, heart rate, or intersegmental vessel area. An asterisk denotes a significant difference from vehicle controls (p<0.05). N = final number of embryos analyzed per treatment.
(TIF)

Figure S6 **Abamectin did not have a concentration dependent effect on any endpoints.** Based on decision criteria defined by Yozzo et al. [8], hashed bars represent concentrations that were not analyzed for potential effects on circulation, pericardial area, heart rate, or intersegmental vessel area. An asterisk denotes a significant difference from vehicle controls (p<0.05). N = final number of embryos analyzed per treatment.
(TIF)

Figure S7 **Propargite did not have a concentration dependent effect on any endpoints.** Based on decision criteria defined by Yozzo et al. [8], hashed bars represent concentrations that were not analyzed for potential effects on circulation, pericardial area, heart rate, or intersegmental vessel area. An asterisk denotes a significant difference from vehicle controls (p<0.05). N = final number of embryos analyzed per treatment.
(TIF)

Figure S8 **Pyraclostrobin did not have a concentration dependent effect on any endpoints.** Based on decision criteria defined by Yozzo et al. [8], hashed bars represent concentrations that were not analyzed for potential effects on circulation, pericardial area, heart rate, or intersegmental vessel area. An asterisk denotes a significant difference from vehicle controls (p<0.05). N = final number of embryos analyzed per treatment.
(TIF)

Figure S9 **(Z, E)-Fenpyroximate did not have a concentration dependent effect on any endpoints.** Based on decision criteria defined by Yozzo et al. [8], hashed bars represent concentrations that were not analyzed for potential effects on circulation, pericardial area, heart rate, or intersegmental vessel area. An asterisk denotes a significant difference from vehicle controls (p<0.05). N = final number of embryos analyzed per treatment.
(TIF)

Figure S10 **Tribufos did not have a concentration dependent effect on any endpoints.** Based on decision criteria defined by Yozzo et al. [8], hashed bars represent concentrations that were not analyzed for potential effects on circulation, pericardial area, heart rate, or intersegmental vessel area. An asterisk denotes a significant difference from vehicle controls (p<0.05). N = final number of embryos analyzed per treatment.
(TIF)

Figure S11 **Famoxadone did not have a concentration dependent effect on any endpoints.** Based on decision criteria defined by Yozzo et al. [8], hashed bars represent concentrations that were not analyzed for potential effects on circulation, pericardial area, heart rate, or intersegmental vessel area. An asterisk denotes a significant difference from vehicle controls (p<0.05). N = final number of embryos analyzed per treatment.
(TIF)

Figure S12 **Fluoxastrobin did not have a concentration dependent effect on any endpoints.** Based on decision criteria defined by Yozzo et al. [8], hashed bars represent concentrations that were not analyzed for potential effects on circulation, pericardial area, heart rate, or intersegmental vessel area. An asterisk denotes a significant difference from vehicle controls (p<0.05). N = final number of embryos analyzed per treatment.
(TIF)

Figure S13 **Pyraflufen-ethyl did not have a concentration dependent effect on any endpoints.** Based on decision criteria defined by Yozzo et al. [8], hashed bars represent concentrations that were not analyzed for potential effects on circulation, pericardial area, heart rate, or intersegmental vessel area. An asterisk denotes a significant difference from vehicle controls (p<0.05). N = final number of embryos analyzed per treatment.
(TIF)

Figure S14 **Trifloxystrobin did not have a concentration dependent effect on any endpoints.** Based on decision criteria defined by Yozzo et al. [8], hashed bars represent concentrations that were not analyzed for potential effects on circulation, pericardial area, heart rate, or intersegmental vessel area. An asterisk denotes a significant difference from vehicle controls (p<0.05). N = final number of embryos analyzed per treatment.
(TIF)

Figure S15 **Dazomet did not have a concentration dependent effect on any endpoints.** Based on decision criteria defined by Yozzo et al. [8], hashed bars represent concentrations that were not analyzed for potential effects on circulation, pericardial area, heart rate, or intersegmental vessel area. An asterisk denotes a significant difference from vehicle controls (p<0.05). N = final number of embryos analyzed per treatment.
(TIF)

Figure S16 **Esfenvalerate did not have a concentration dependent effect on any endpoints.** Based on decision criteria defined by Yozzo et al. [8], hashed bars represent concentrations that were not analyzed for potential effects on circulation, pericardial area, heart rate, or intersegmental vessel area. An asterisk denotes a significant difference from vehicle controls (p<0.05). N = final number of embryos analyzed per treatment.
(TIF)

Figure S17 **Chlorothalonil did not have a concentration dependent effect on any endpoints.** Based on decision criteria defined by Yozzo et al. [8], hashed bars represent concentrations that were not analyzed for potential effects on circulation, pericardial area, heart rate, or intersegmental vessel area. An asterisk denotes a significant difference from vehicle controls (p<0.05). N = final number of embryos analyzed per treatment.
(TIF)

Figure S18 **Lactofen did not have a concentration dependent effect on any endpoints.** Based on decision criteria defined by Yozzo et al. [8], hashed bars represent concentrations that were not analyzed for potential effects on circulation, pericardial area, heart rate, or intersegmental vessel area. An asterisk denotes a significant difference from vehicle controls (p<0.05). N = final number of embryos analyzed per treatment.
(TIF)

Figure S19 **Clodinafop-propargyl did not have a concentration dependent effect on any endpoints.** Based on decision criteria defined by Yozzo et al. [8], hashed bars represent concentrations that were not analyzed for potential effects on circulation, pericardial area, heart rate, or intersegmental vessel area. An asterisk denotes a significant difference from vehicle controls (p<0.05). N = final number of embryos analyzed per treatment.
(TIF)

Figure S20 **Tebufenpyrad did not have a concentration dependent effect on any endpoints.** Based on decision criteria defined by Yozzo et al. [8], hashed bars represent concentrations that were not analyzed for potential effects on circulation, pericardial area, heart rate, or intersegmental vessel area. An asterisk denotes a significant difference from vehicle controls (p<0.05). N = final number of embryos analyzed per treatment.
(TIF)

Figure S21 **Fenpropathrin did not have a concentration dependent effect on any endpoints.** Based on decision criteria defined by Yozzo et al. [8], hashed bars represent concentrations that were not analyzed for potential effects on circulation, pericardial area, heart rate, or intersegmental vessel area. An asterisk denotes a significant difference from vehicle controls (p<0.05). N = final number of embryos analyzed per treatment.
(TIF)

Figure S22 **Cyfluthrin did not have a concentration dependent effect on any endpoints.** Based on decision criteria defined by Yozzo et al. [8], hashed bars represent concentrations that were not analyzed for potential effects on circulation, pericardial area, heart rate, or intersegmental vessel area. An asterisk denotes a significant difference from vehicle controls (p<0.05). N = final number of embryos analyzed per treatment.
(TIF)

Figure S23 **Indoxacarb did not have a concentration dependent effect on any endpoints.** Based on decision criteria defined by Yozzo et al. [8], hashed bars represent concentrations that were not analyzed for potential effects on circulation, pericardial area, heart rate, or intersegmental vessel area. An asterisk denotes a significant difference from vehicle controls (p<0.05). N = final number of embryos analyzed per treatment.
(TIF)

Figure S24 **Chlorpyrifos (ethyl) oxon did not have a concentration dependent effect on any endpoints.** Based on decision criteria defined by Yozzo et al. [8], hashed bars represent concentrations that were not analyzed for potential effects on circulation, pericardial area, heart rate, or intersegmental vessel area. An asterisk denotes a significant difference from vehicle controls (p<0.05). N = final number of embryos analyzed per treatment.
(TIF)

Figure S25 **Flufenpyr-ethyl did not have a concentration dependent effect on any endpoints.** Based on decision criteria defined by Yozzo et al. [8], hashed bars represent concentrations that were not analyzed for potential effects on circulation, pericardial area, heart rate, or intersegmental vessel area. An asterisk denotes a significant difference from vehicle controls (p<0.05). N = final number of embryos analyzed per treatment.
(TIF)

Figure S26 **Carfentrazone-ethyl did not have a concentration dependent effect on any endpoints.** Based on decision criteria defined by Yozzo et al. [8], hashed bars represent concentrations that were not analyzed for potential effects on circulation, pericardial area, heart rate, or intersegmental vessel area. An asterisk denotes a significant difference from vehicle controls (p<0.05). N = final number of embryos analyzed per treatment.
(TIF)

Figure S27 **Flumiclorac-pentyl did not have a concentration dependent effect on any endpoints.** Based on decision criteria defined by Yozzo et al. [8], hashed bars represent concentrations that were not analyzed for potential effects on circulation, pericardial area, heart rate, or intersegmental vessel area. An asterisk denotes a significant difference from vehicle controls (p<0.05). N = final number of embryos analyzed per treatment.
(TIF)

Figure S28 **Oxadiazon did not have a concentration dependent effect on any endpoints.** Based on decision criteria defined by Yozzo et al. [8], hashed bars represent concentrations that were not analyzed for potential effects on circulation, pericardial area, heart rate, or intersegmental vessel area. An asterisk denotes a significant difference from vehicle controls (p<0.05). N = final number of embryos analyzed per treatment.
(TIF)

Figure S29 **Flumioxazin did not have a concentration dependent effect on any endpoints.** Based on decision criteria defined by Yozzo et al. [8], hashed bars represent concentrations that were not analyzed for potential effects on circulation, pericardial area, heart rate, or intersegmental vessel area. An asterisk denotes a significant difference from vehicle controls (p<0.05). N = final number of embryos analyzed per treatment.
(TIF)

Figure S30 **Oxyfluorfen did not have a concentration dependent effect on any endpoints.** Based on decision criteria defined by Yozzo et al. [8], hashed bars represent concentrations that were not analyzed for potential effects on circulation, pericardial area, heart rate, or intersegmental vessel area. An asterisk denotes a significant difference from vehicle controls (p<0.05). N = final number of embryos analyzed per treatment.
(TIF)

Figure S31 **Sulfentrazone did not have a concentration dependent effect on any endpoints.** Based on decision criteria defined by Yozzo et al. [8], hashed bars represent concentrations that were not analyzed for potential effects on circulation, pericardial area, heart rate, or intersegmental vessel area. An asterisk denotes a significant difference from vehicle controls (p<0.05). N = final number of embryos analyzed per treatment.
(TIF)

Figure S32 **Acifluorfen did not have a concentration dependent effect on any endpoints.** Based on decision criteria defined by Yozzo et al. [8], hashed bars represent concentrations that were not analyzed for potential effects on circulation, pericardial area, heart rate, or intersegmental vessel area. An asterisk denotes a significant difference from vehicle controls (p<0.05). N = final number of embryos analyzed per treatment.
(TIF)

Table S1 **Chemical name, chemical formula, CAS registry number, vendor, and purity of all chemicals screened.**
(PDF)

Video S1 **Circulation of blood through a 72-hpf zebrafish embryo exposed to 3.125 µM butafenacil.**
(AVI)

Video S2 **Circulation of blood through a 72-hpf zebrafish embryo exposed to vehicle control (0.1% DMSO).**
(AVI)

Dataset S1 **Microsoft Excel spreadsheets containing raw data for all chemical screening assays.**
(ZIP)
