# Supplementary figures and images for: High-Content Screening in Zebrafish Embryos Identifies Butafenacil as a Potent Inducer of Anemia
Source: PLoS One. 2014 Aug 4;9(8):e104190. doi: 10.1371/journal.pone.0104190 (PMC4121296; doi:10.1371/journal.pone.0104190)

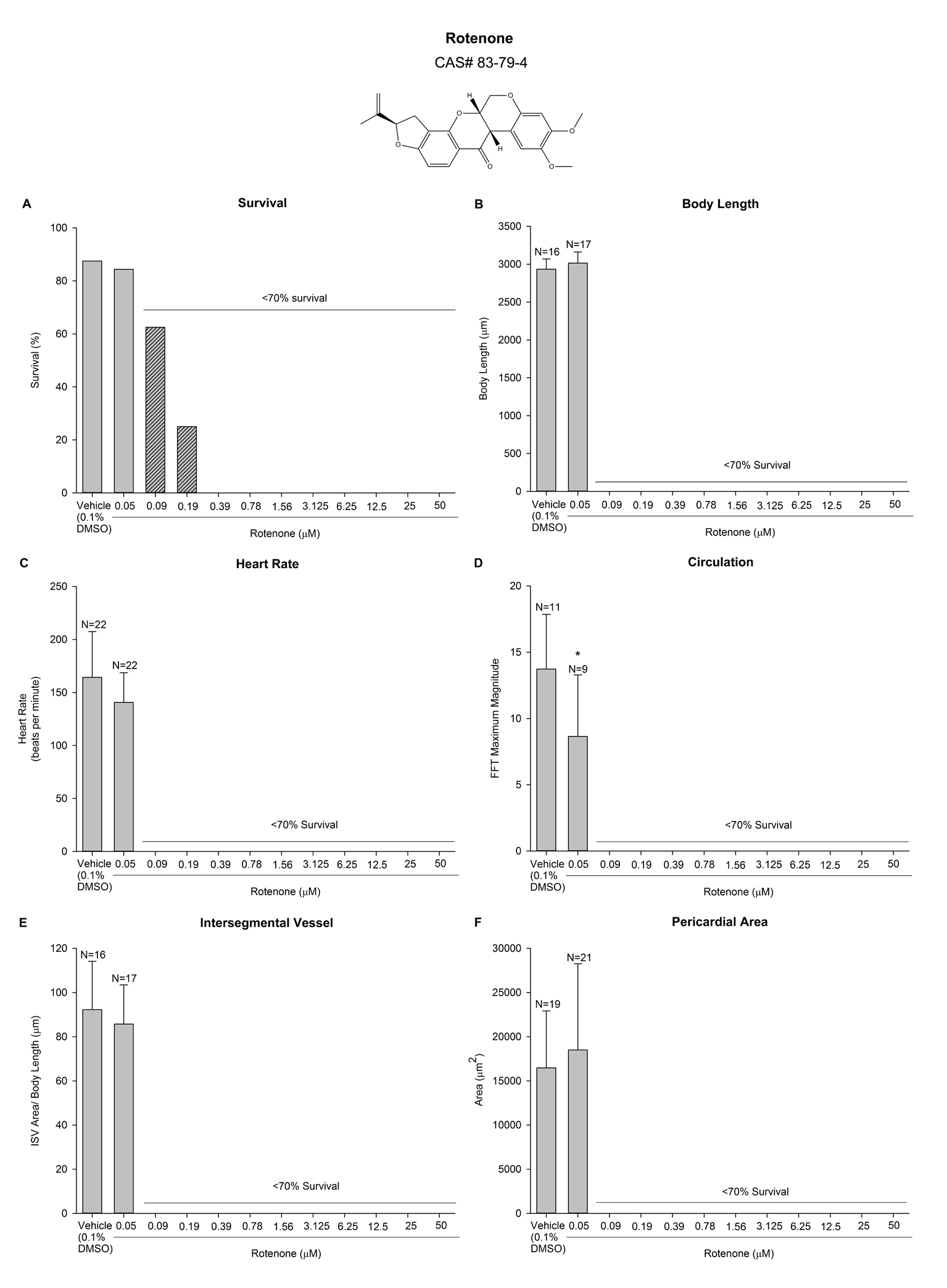

Supplement: Figure S1 — Rotenone did not have a concentration dependent effect on any endpoints. Based on decision criteria defined by Yozzo et al. [8], hashed bars represent concentrations that were not analyzed for potential effects on circulation, pericardial area, heart rate, or intersegmental vessel area. An asterisk denotes a significant difference from vehicle controls (p<0.05). N = final number of embryos analyzed per treatment. (TIF) [file pone.0104190.s001.tif]

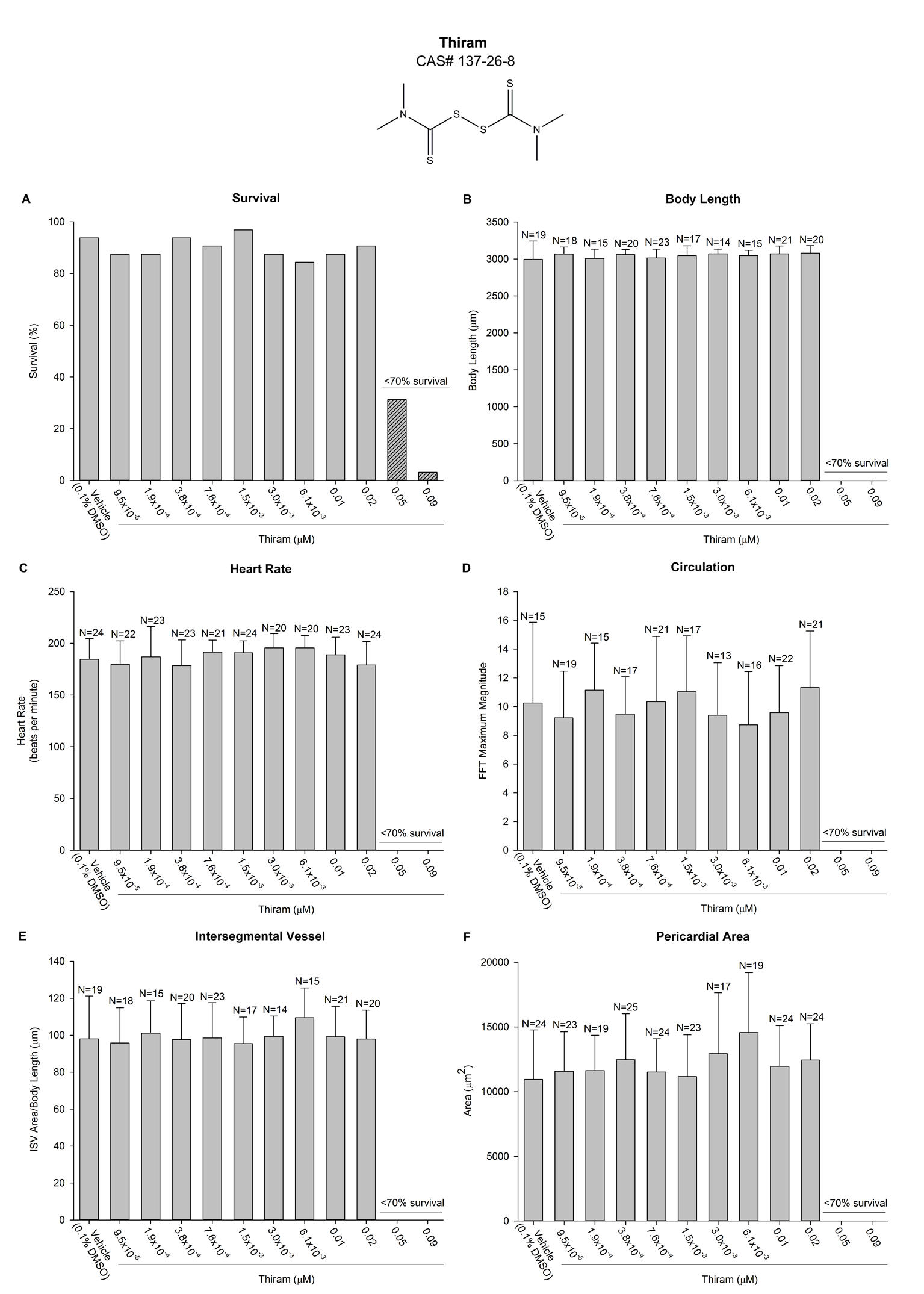

Supplement: Figure S2 — Thiram did not have a concentration dependent effect on any endpoints. Based on decision criteria defined by Yozzo et al. [8], hashed bars represent concentrations that were not analyzed for potential effects on circulation, pericardial area, heart rate, or intersegmental vessel area. An asterisk denotes a significant difference from vehicle controls (p<0.05). N = final number of embryos analyzed per treatment. (TIF) [file pone.0104190.s002.tif]

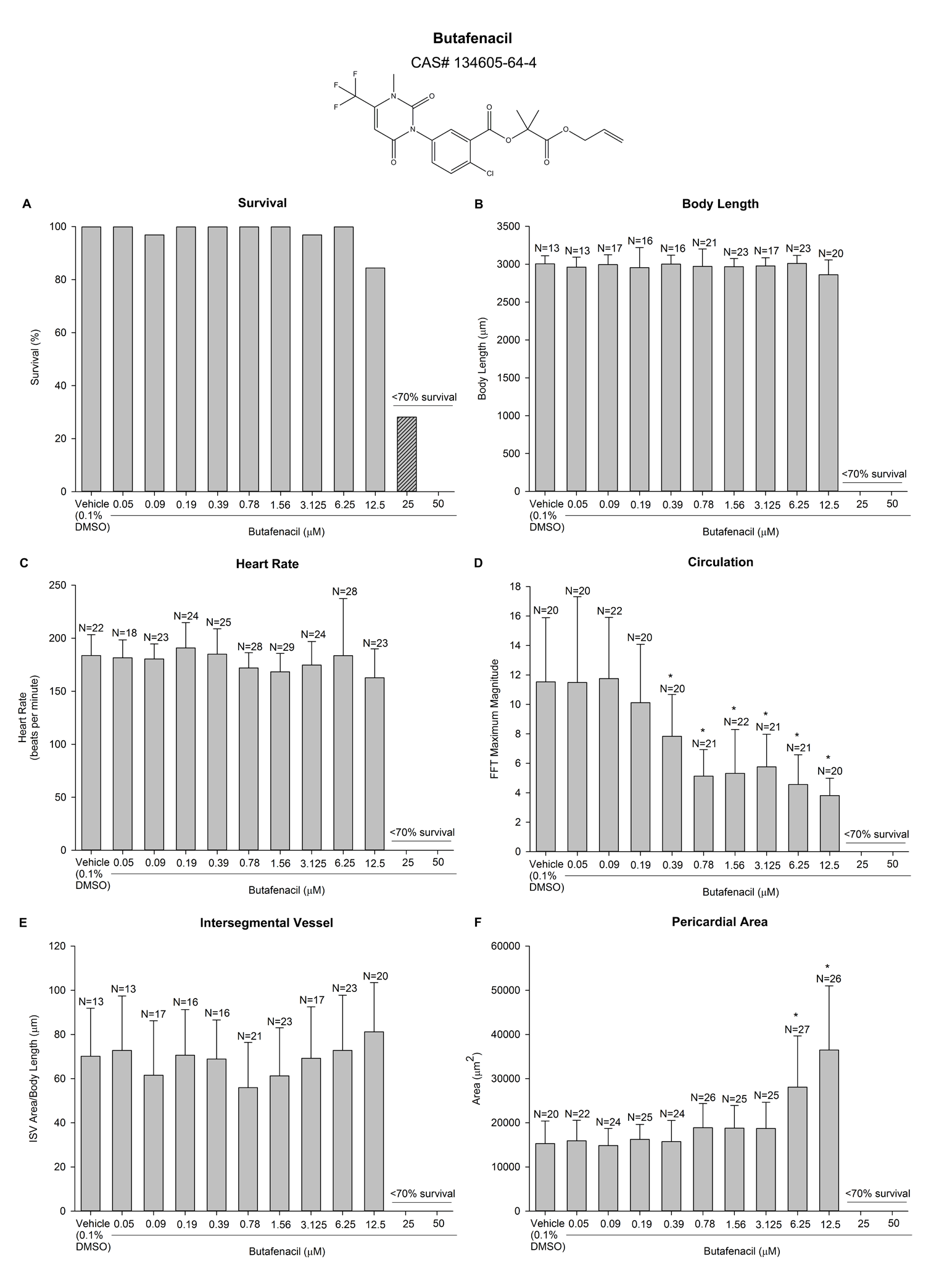

Supplement: Figure S3 — Butafenacil significantly decreased circulation without having a concentration dependent effect on any other endpoint. Based on decision criteria defined by Yozzo et al. [8], hashed bars represent concentrations that were not analyzed for potential effects on circulation, pericardial area, heart rate, or intersegmental vessel area. An asterisk denotes a significant difference from vehicle controls (p<0.05). N = final number of embryos analyzed per treatment. (TIF) [file pone.0104190.s003.tif]

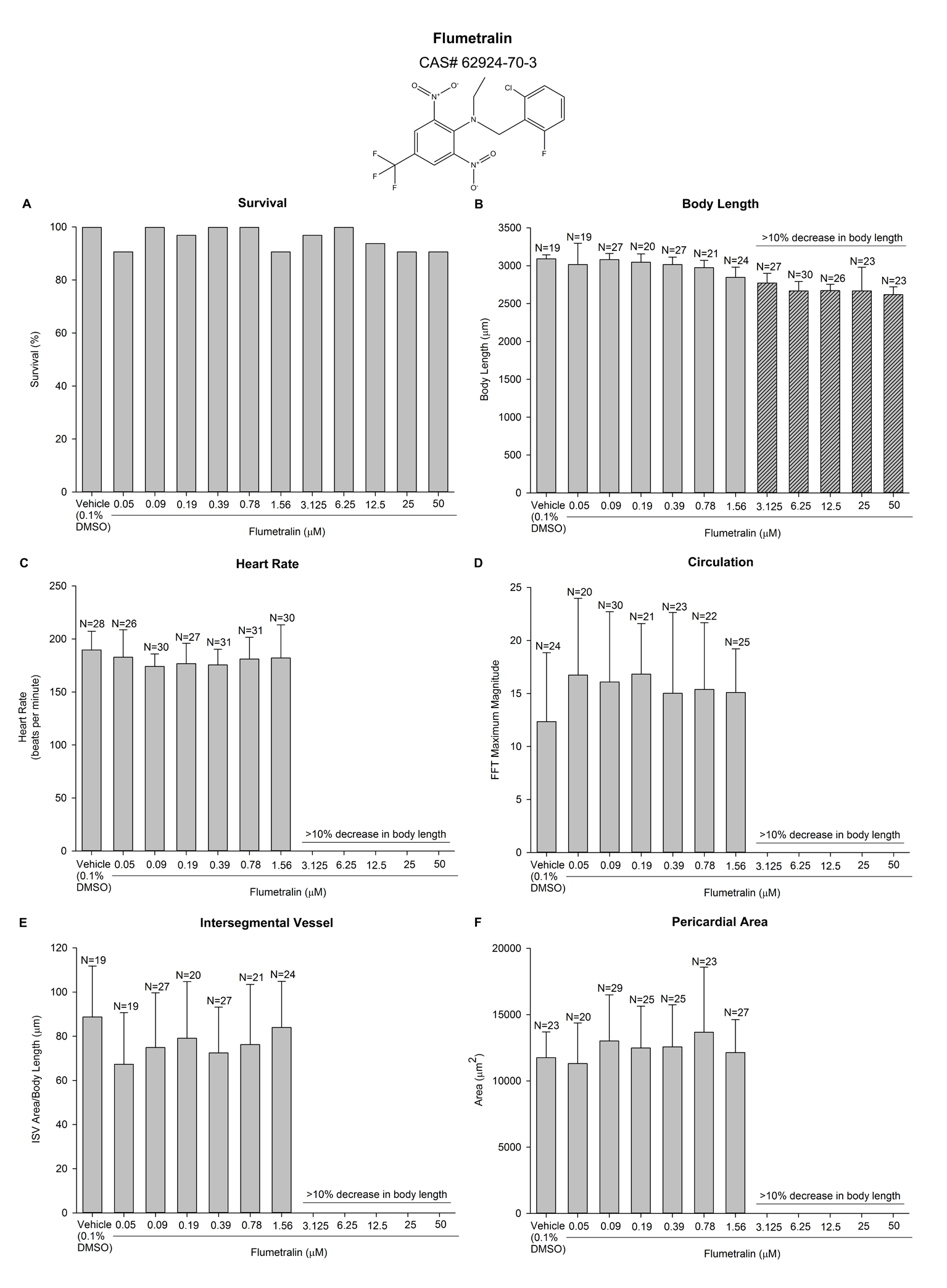

Supplement: Figure S4 — Flumetralin did not have a concentration dependent effect on any endpoints. Based on decision criteria defined by Yozzo et al. [8], hashed bars represent concentrations that were not analyzed for potential effects on circulation, pericardial area, heart rate, or intersegmental vessel area. An asterisk denotes a significant difference from vehicle controls (p<0.05). N = final number of embryos analyzed per treatment. (TIF) [file pone.0104190.s004.tif]

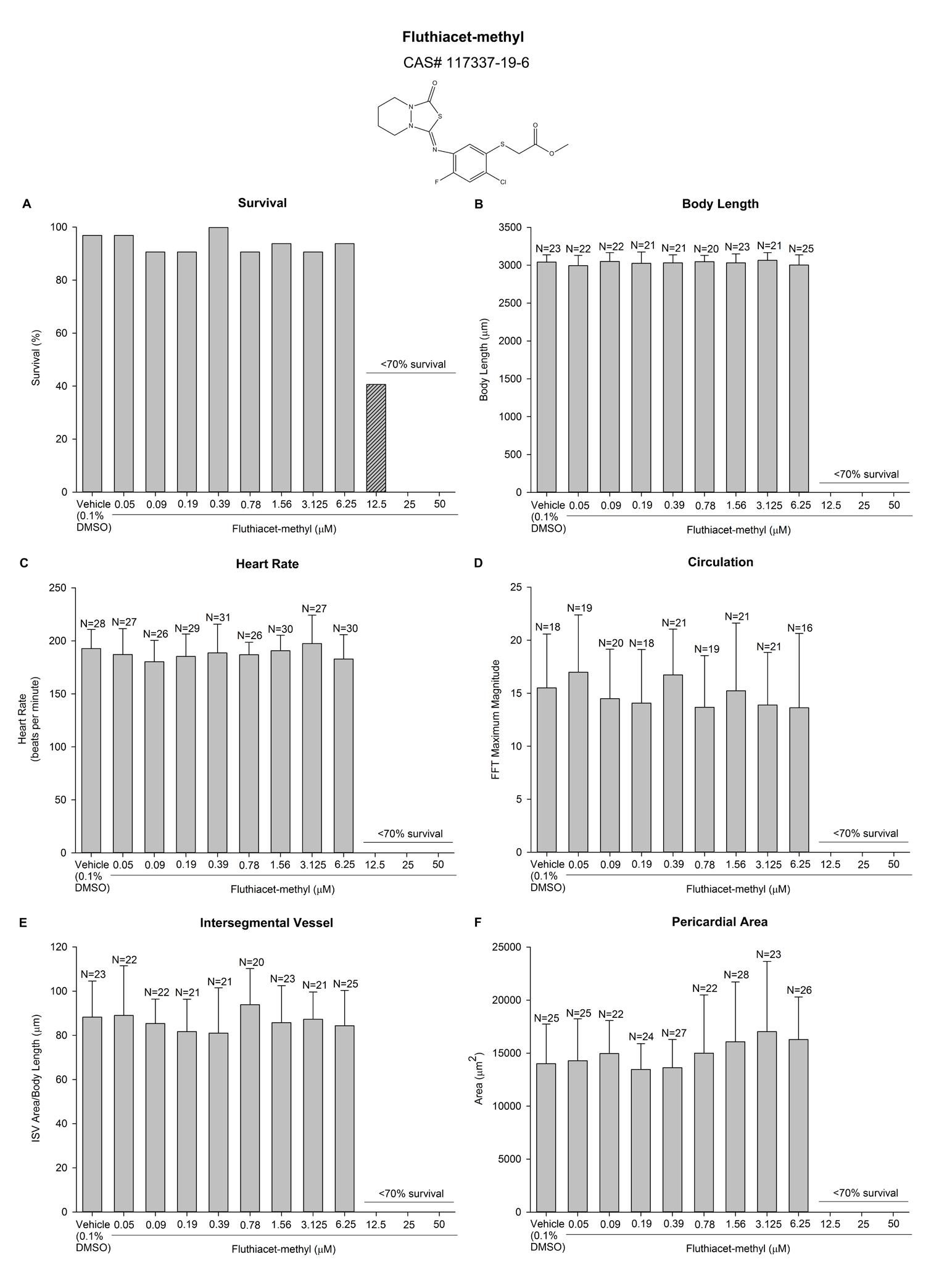

Supplement: Figure S5 — Fluthiacet-methyl did not have a concentration dependent effect on any endpoints. Based on decision criteria defined by Yozzo et al. [8], hashed bars represent concentrations that were not analyzed for potential effects on circulation, pericardial area, heart rate, or intersegmental vessel area. An asterisk denotes a significant difference from vehicle controls (p<0.05). N = final number of embryos analyzed per treatment. (TIF) [file pone.0104190.s005.tif]

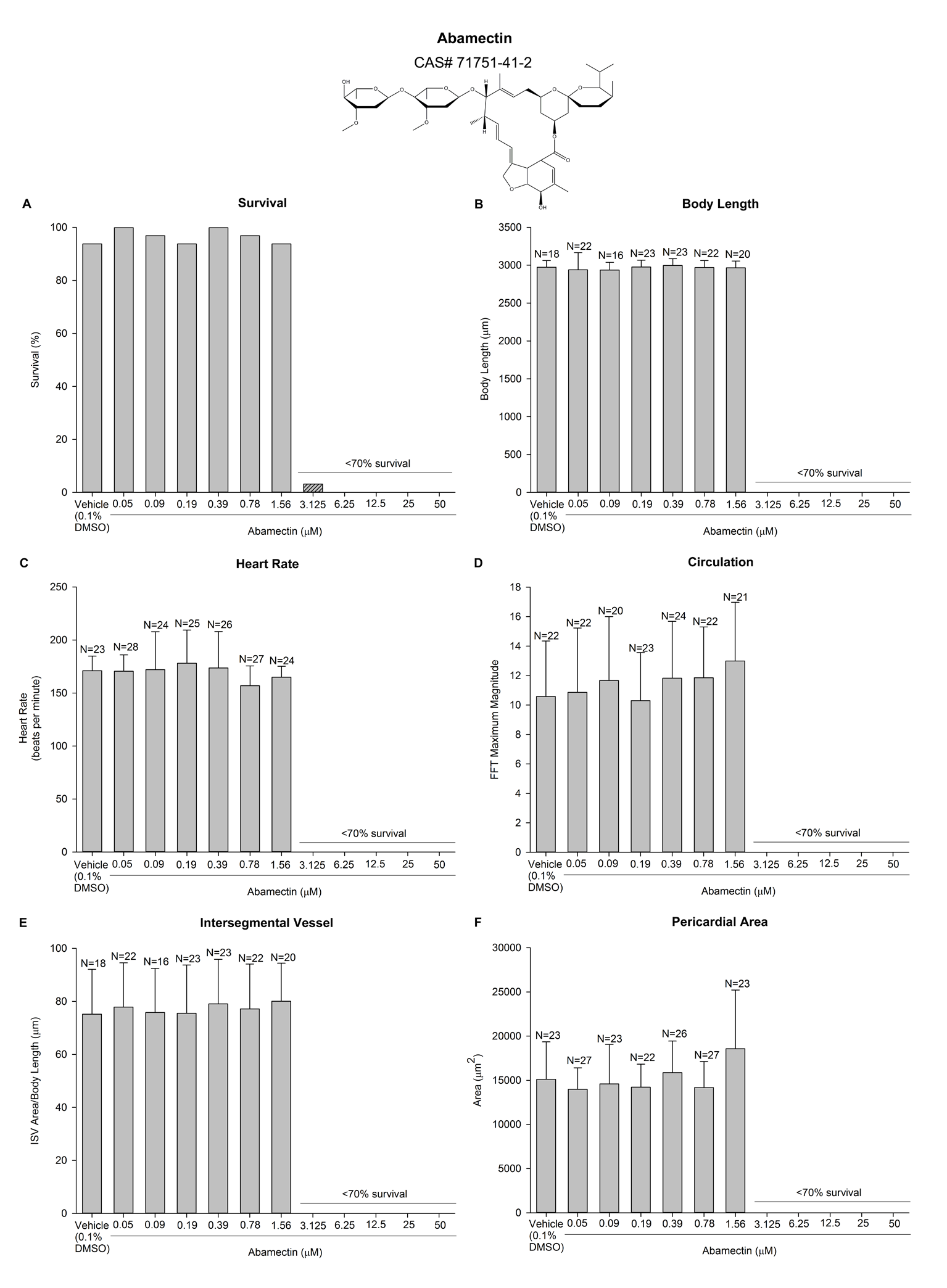

Supplement: Figure S6 — Abamectin did not have a concentration dependent effect on any endpoints. Based on decision criteria defined by Yozzo et al. [8], hashed bars represent concentrations that were not analyzed for potential effects on circulation, pericardial area, heart rate, or intersegmental vessel area. An asterisk denotes a significant difference from vehicle controls (p<0.05). N = final number of embryos analyzed per treatment. (TIF) [file pone.0104190.s006.tif]

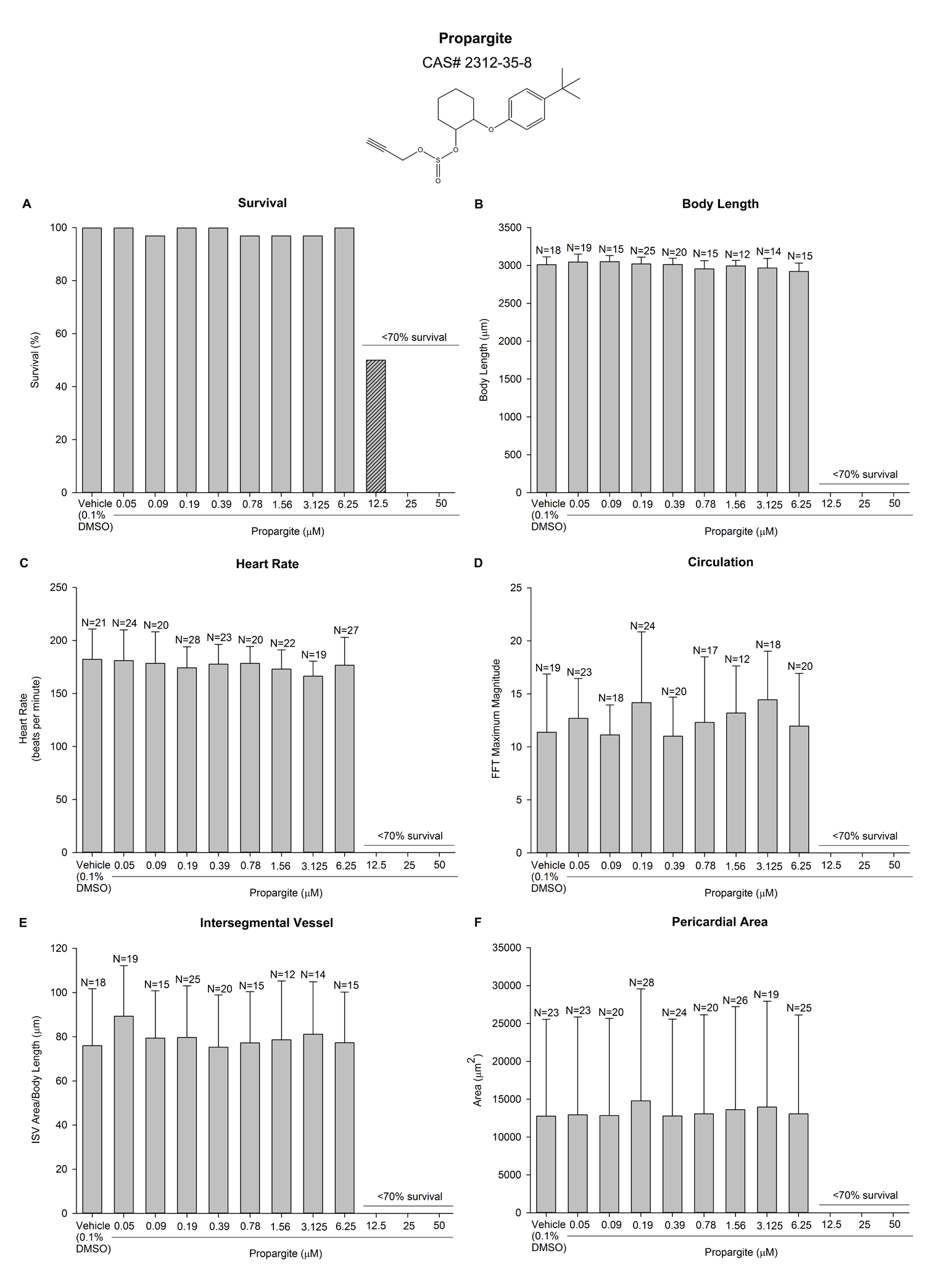

Supplement: Figure S7 — Propargite did not have a concentration dependent effect on any endpoints. Based on decision criteria defined by Yozzo et al. [8], hashed bars represent concentrations that were not analyzed for potential effects on circulation, pericardial area, heart rate, or intersegmental vessel area. An asterisk denotes a significant difference from vehicle controls (p<0.05). N = final number of embryos analyzed per treatment. (TIF) [file pone.0104190.s007.tif]

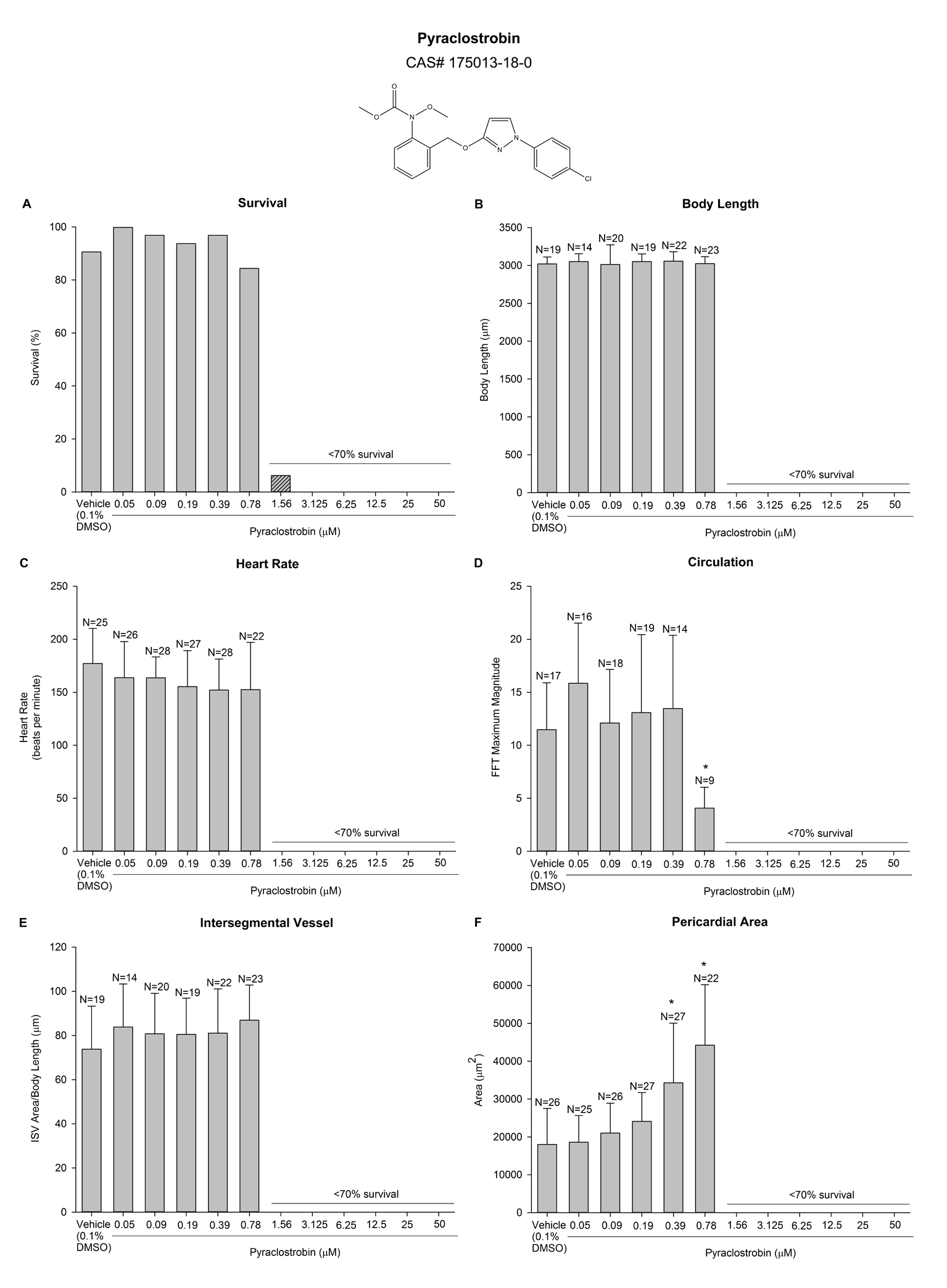

Supplement: Figure S8 — Pyraclostrobin did not have a concentration dependent effect on any endpoints. Based on decision criteria defined by Yozzo et al. [8], hashed bars represent concentrations that were not analyzed for potential effects on circulation, pericardial area, heart rate, or intersegmental vessel area. An asterisk denotes a significant difference from vehicle controls (p<0.05). N = final number of embryos analyzed per treatment. (TIF) [file pone.0104190.s008.tif]

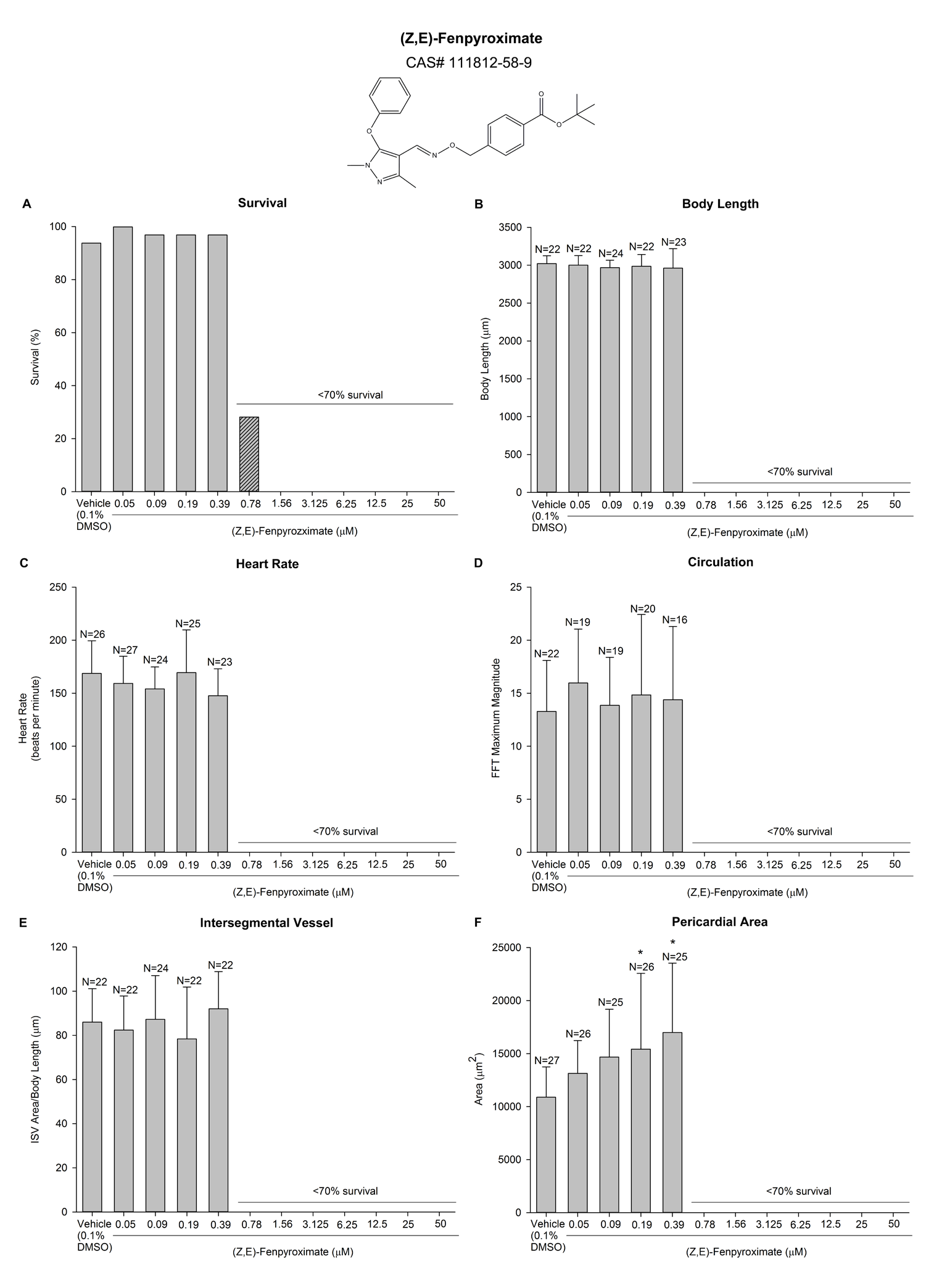

Supplement: Figure S9 — (Z, E)-Fenpyroximate did not have a concentration dependent effect on any endpoints. Based on decision criteria defined by Yozzo et al. [8], hashed bars represent concentrations that were not analyzed for potential effects on circulation, pericardial area, heart rate, or intersegmental vessel area. An asterisk denotes a significant difference from vehicle controls (p<0.05). N = final number of embryos analyzed per treatment. (TIF) [file pone.0104190.s009.tif]

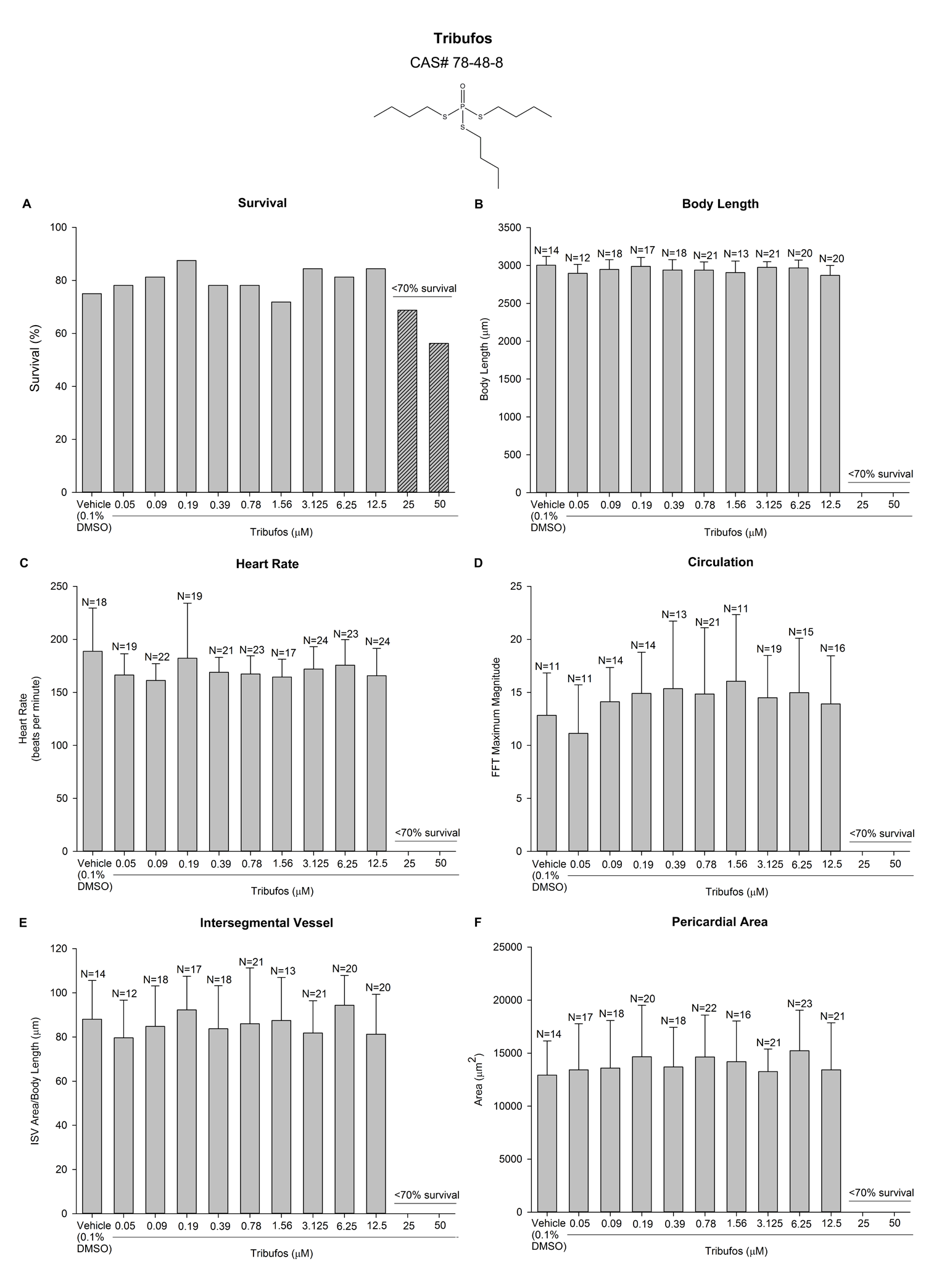

Supplement: Figure S10 — Tribufos did not have a concentration dependent effect on any endpoints. Based on decision criteria defined by Yozzo et al. [8], hashed bars represent concentrations that were not analyzed for potential effects on circulation, pericardial area, heart rate, or intersegmental vessel area. An asterisk denotes a significant difference from vehicle controls (p<0.05). N = final number of embryos analyzed per treatment. (TIF) [file pone.0104190.s010.tif]

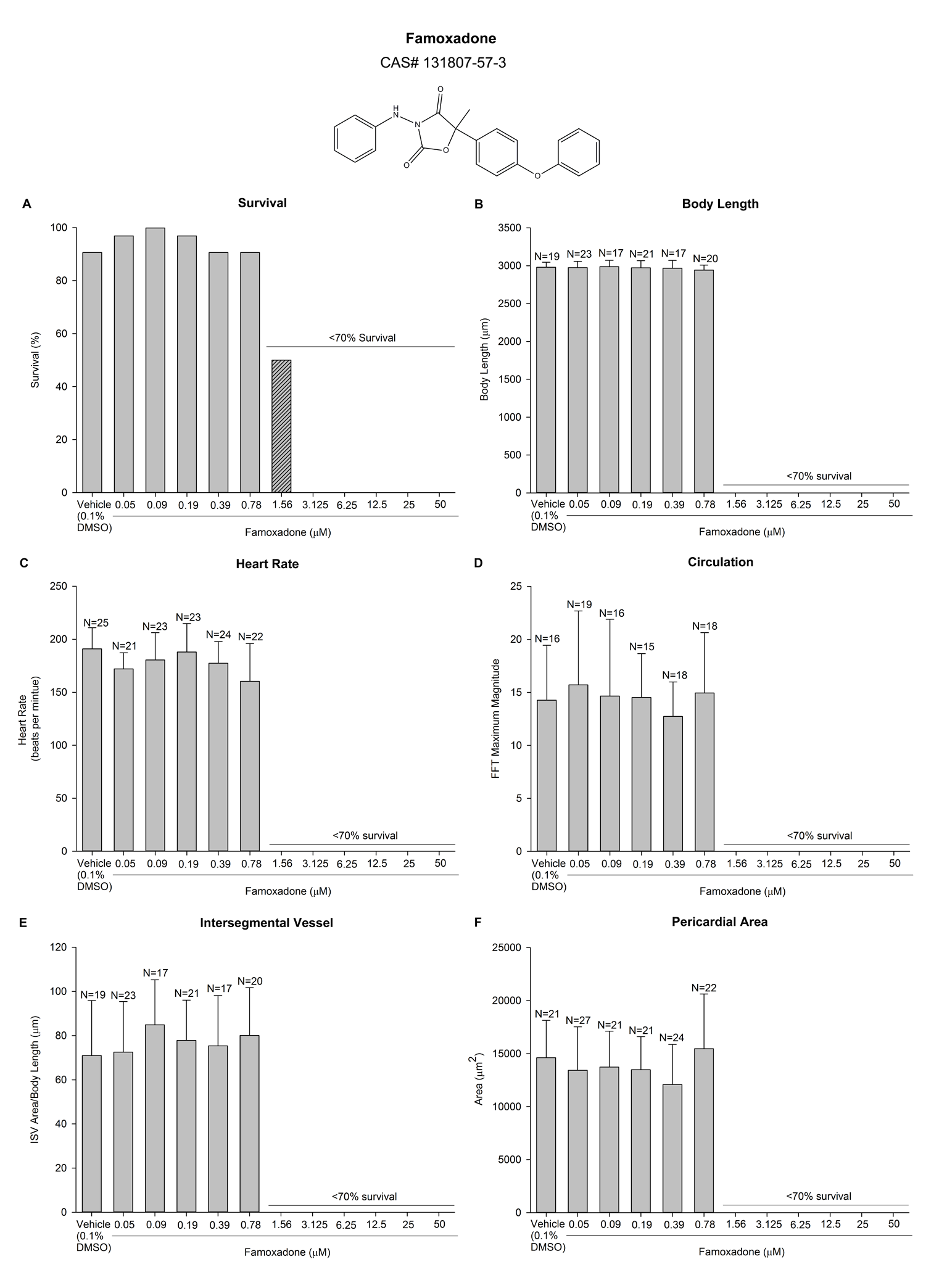

Supplement: Figure S11 — Famoxadone did not have a concentration dependent effect on any endpoints. Based on decision criteria defined by Yozzo et al. [8], hashed bars represent concentrations that were not analyzed for potential effects on circulation, pericardial area, heart rate, or intersegmental vessel area. An asterisk denotes a significant difference from vehicle controls (p<0.05). N = final number of embryos analyzed per treatment. (TIF) [file pone.0104190.s011.tif]

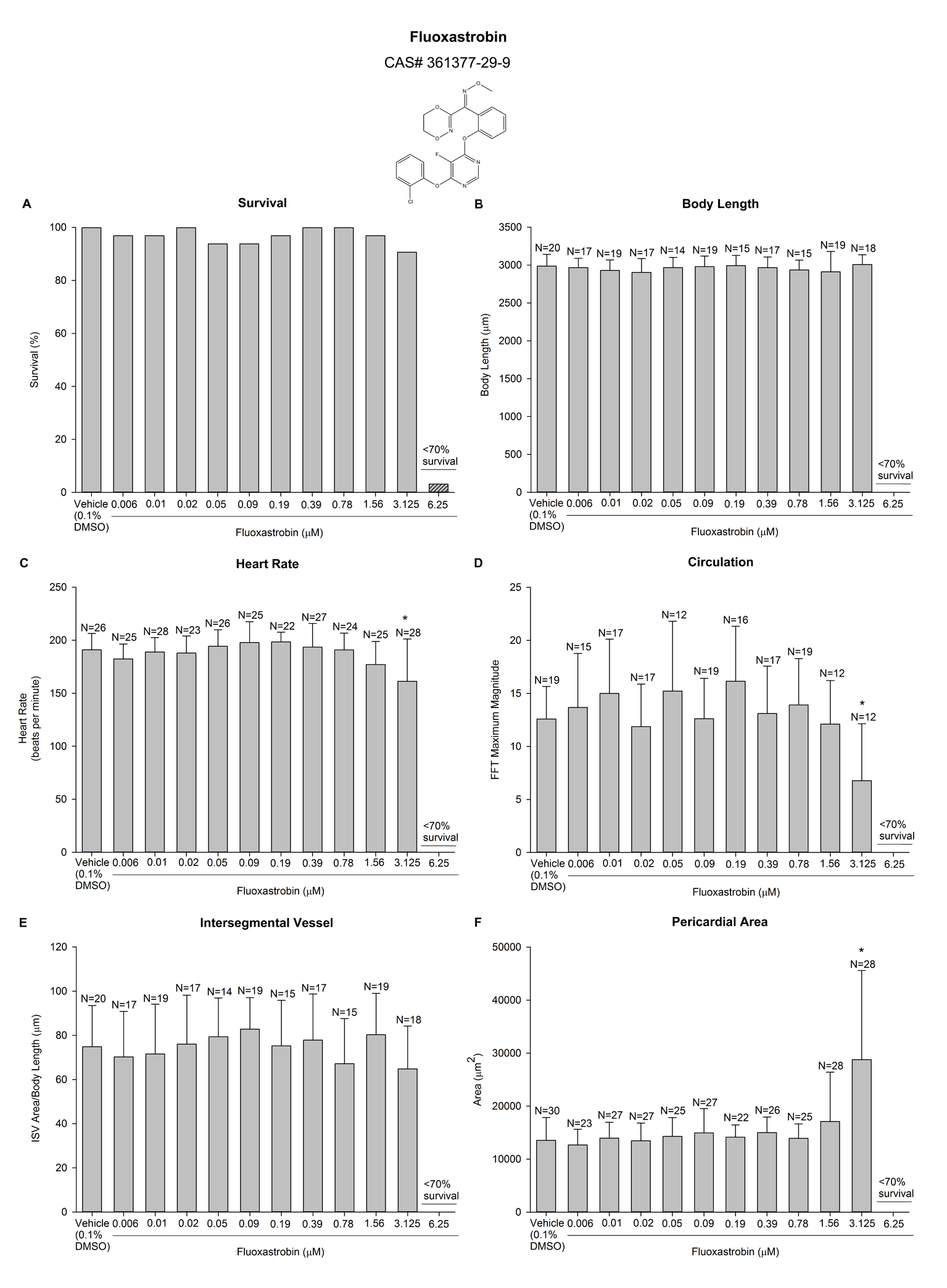

Supplement: Figure S12 — Fluoxastrobin did not have a concentration dependent effect on any endpoints. Based on decision criteria defined by Yozzo et al. [8], hashed bars represent concentrations that were not analyzed for potential effects on circulation, pericardial area, heart rate, or intersegmental vessel area. An asterisk denotes a significant difference from vehicle controls (p<0.05). N = final number of embryos analyzed per treatment. (TIF) [file pone.0104190.s012.tif]

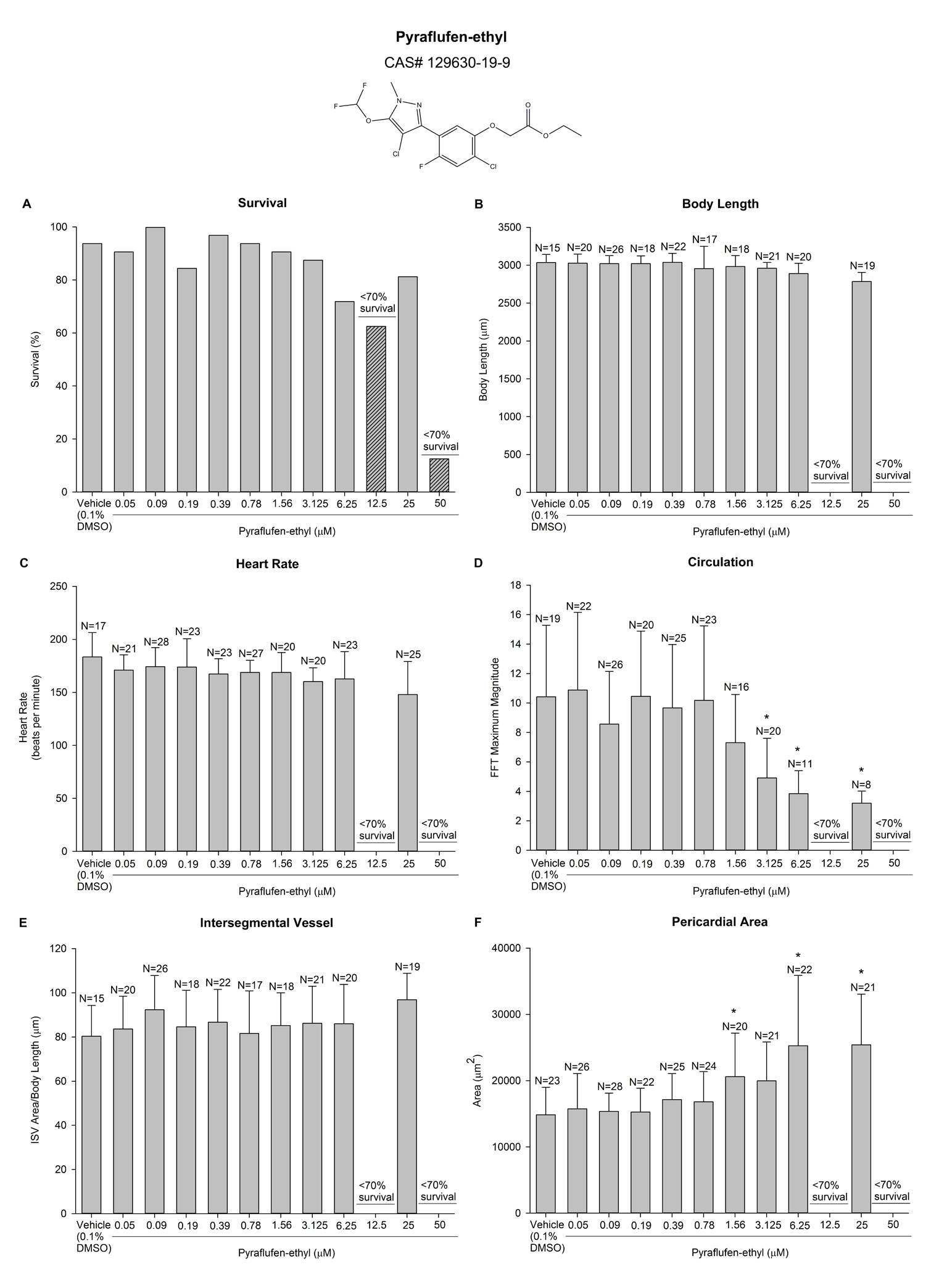

Supplement: Figure S13 — Pyraflufen-ethyl did not have a concentration dependent effect on any endpoints. Based on decision criteria defined by Yozzo et al. [8], hashed bars represent concentrations that were not analyzed for potential effects on circulation, pericardial area, heart rate, or intersegmental vessel area. An asterisk denotes a significant difference from vehicle controls (p<0.05). N = final number of embryos analyzed per treatment. (TIF) [file pone.0104190.s013.tif]

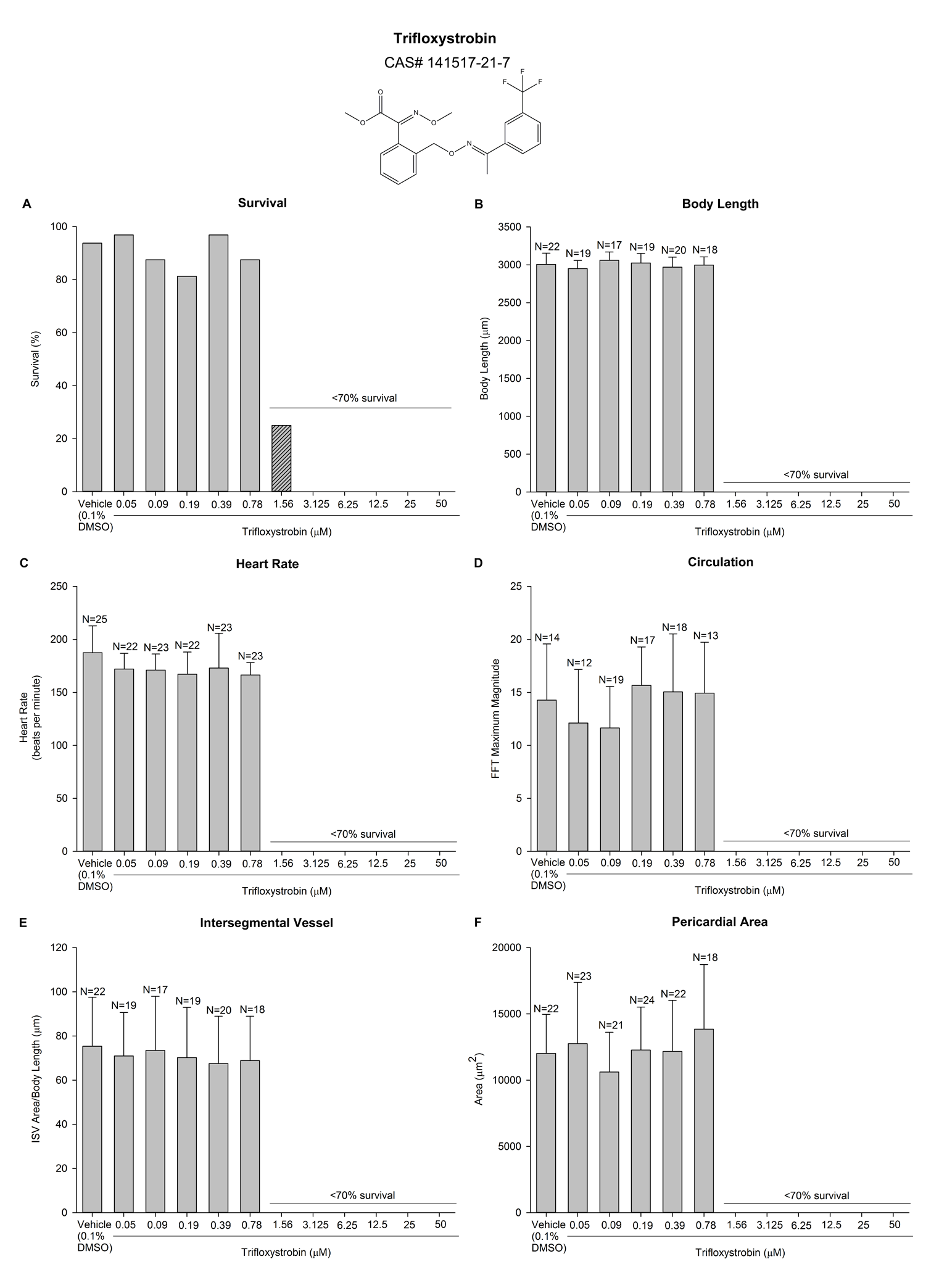

Supplement: Figure S14 — Trifloxystrobin did not have a concentration dependent effect on any endpoints. Based on decision criteria defined by Yozzo et al. [8], hashed bars represent concentrations that were not analyzed for potential effects on circulation, pericardial area, heart rate, or intersegmental vessel area. An asterisk denotes a significant difference from vehicle controls (p<0.05). N = final number of embryos analyzed per treatment. (TIF) [file pone.0104190.s014.tif]

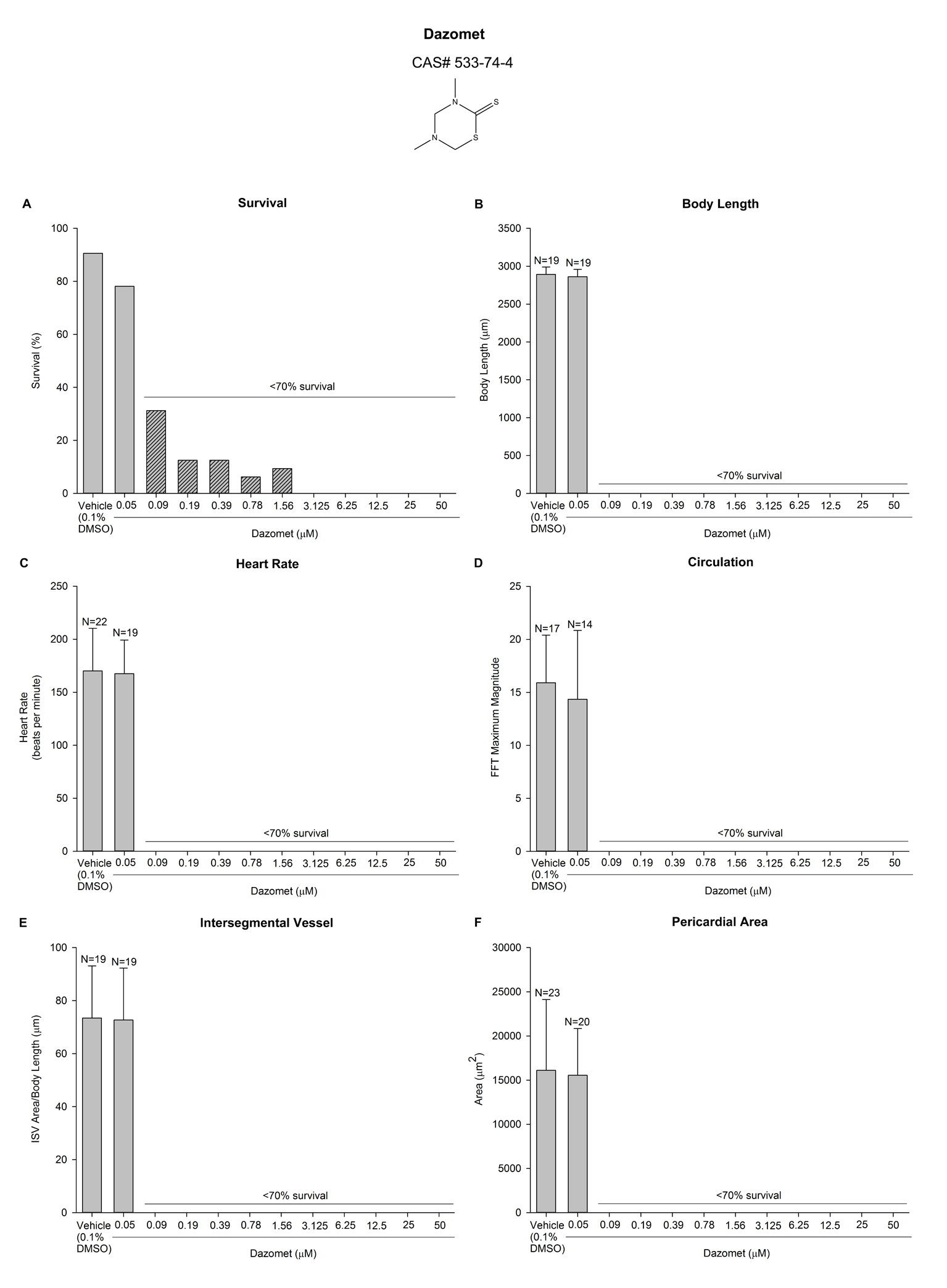

Supplement: Figure S15 — Dazomet did not have a concentration dependent effect on any endpoints. Based on decision criteria defined by Yozzo et al. [8], hashed bars represent concentrations that were not analyzed for potential effects on circulation, pericardial area, heart rate, or intersegmental vessel area. An asterisk denotes a significant difference from vehicle controls (p<0.05). N = final number of embryos analyzed per treatment. (TIF) [file pone.0104190.s015.tif]

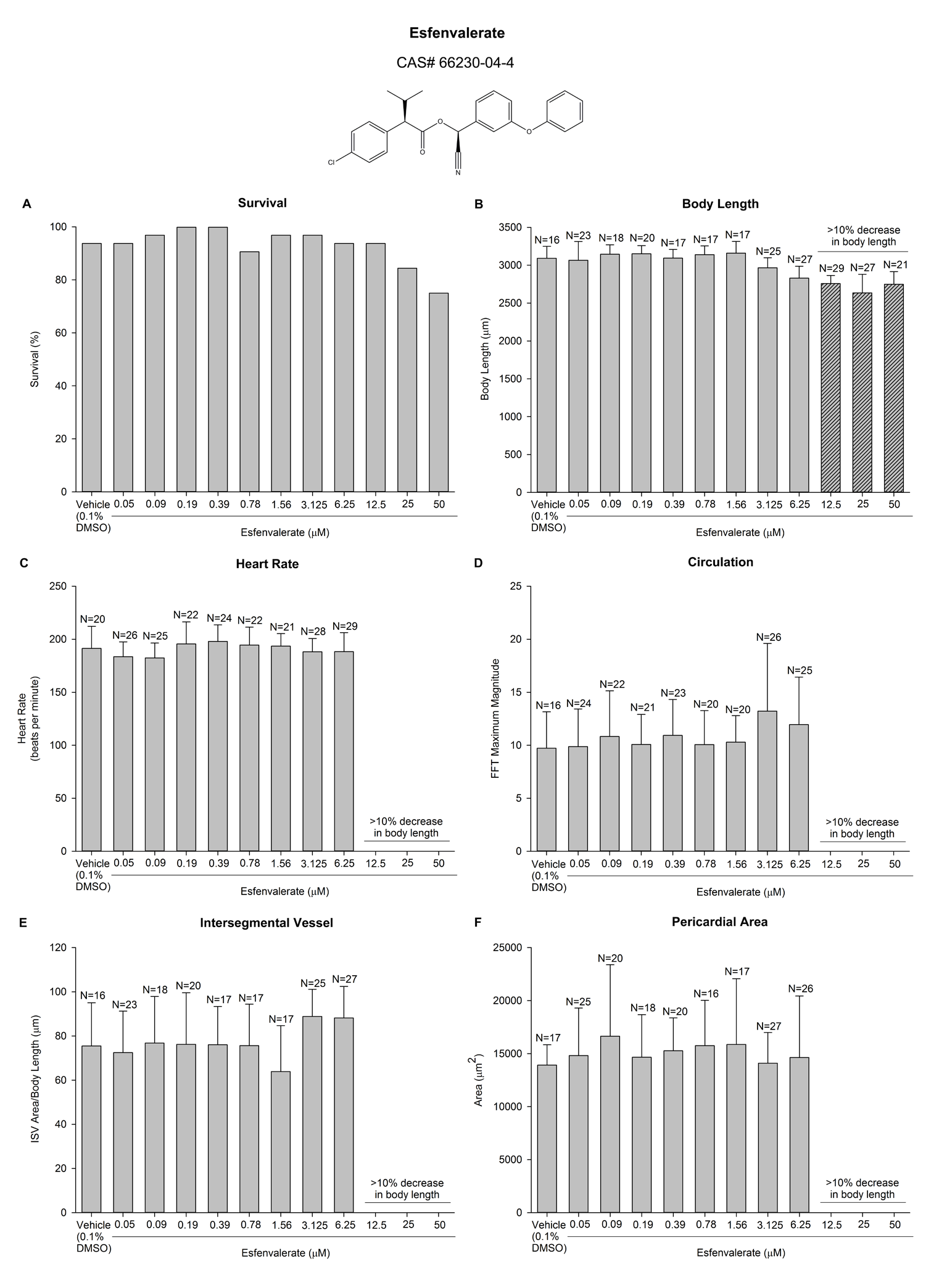

Supplement: Figure S16 — Esfenvalerate did not have a concentration dependent effect on any endpoints. Based on decision criteria defined by Yozzo et al. [8], hashed bars represent concentrations that were not analyzed for potential effects on circulation, pericardial area, heart rate, or intersegmental vessel area. An asterisk denotes a significant difference from vehicle controls (p<0.05). N = final number of embryos analyzed per treatment. (TIF) [file pone.0104190.s016.tif]

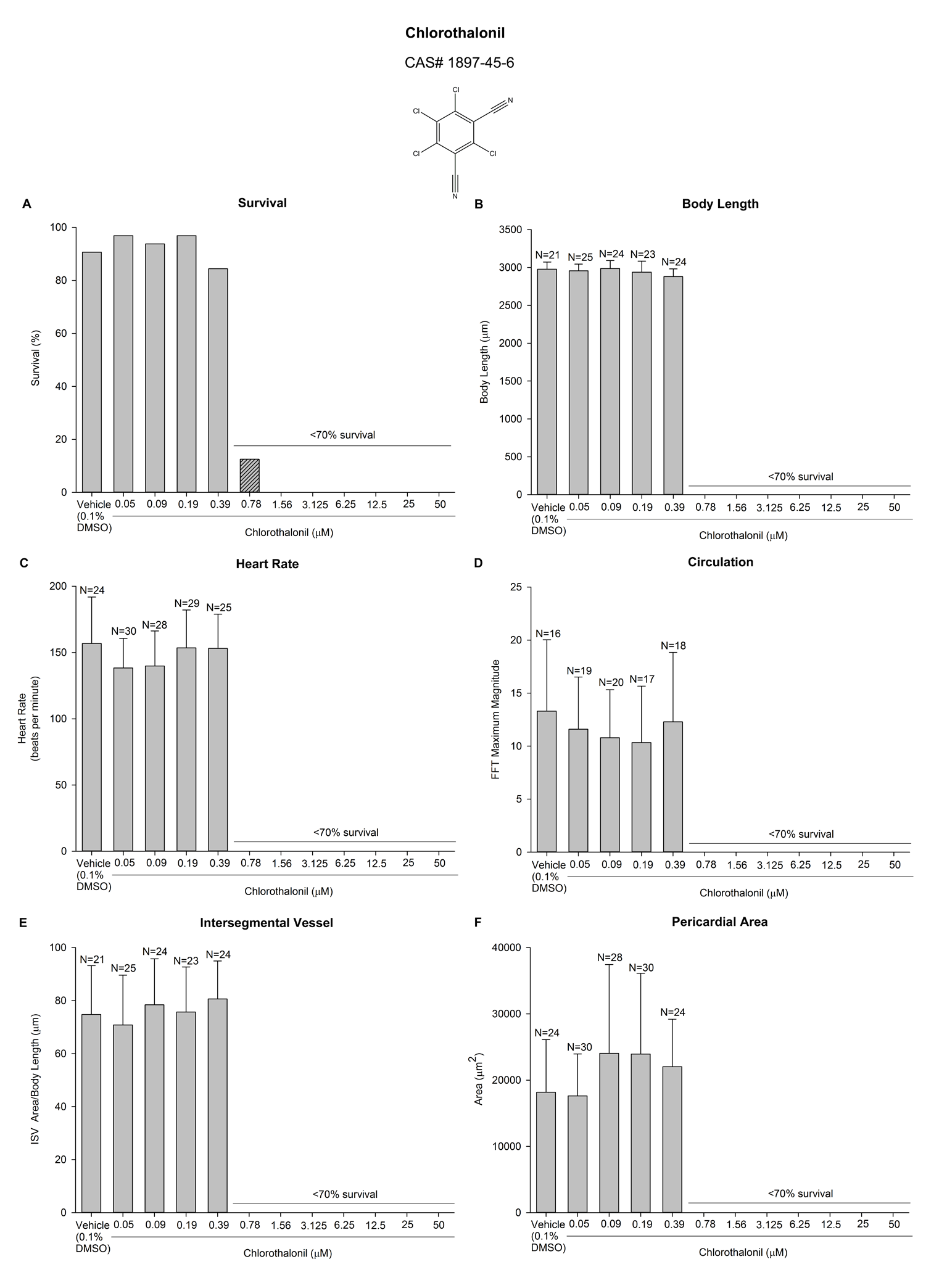

Supplement: Figure S17 — Chlorothalonil did not have a concentration dependent effect on any endpoints. Based on decision criteria defined by Yozzo et al. [8], hashed bars represent concentrations that were not analyzed for potential effects on circulation, pericardial area, heart rate, or intersegmental vessel area. An asterisk denotes a significant difference from vehicle controls (p<0.05). N = final number of embryos analyzed per treatment. (TIF) [file pone.0104190.s017.tif]

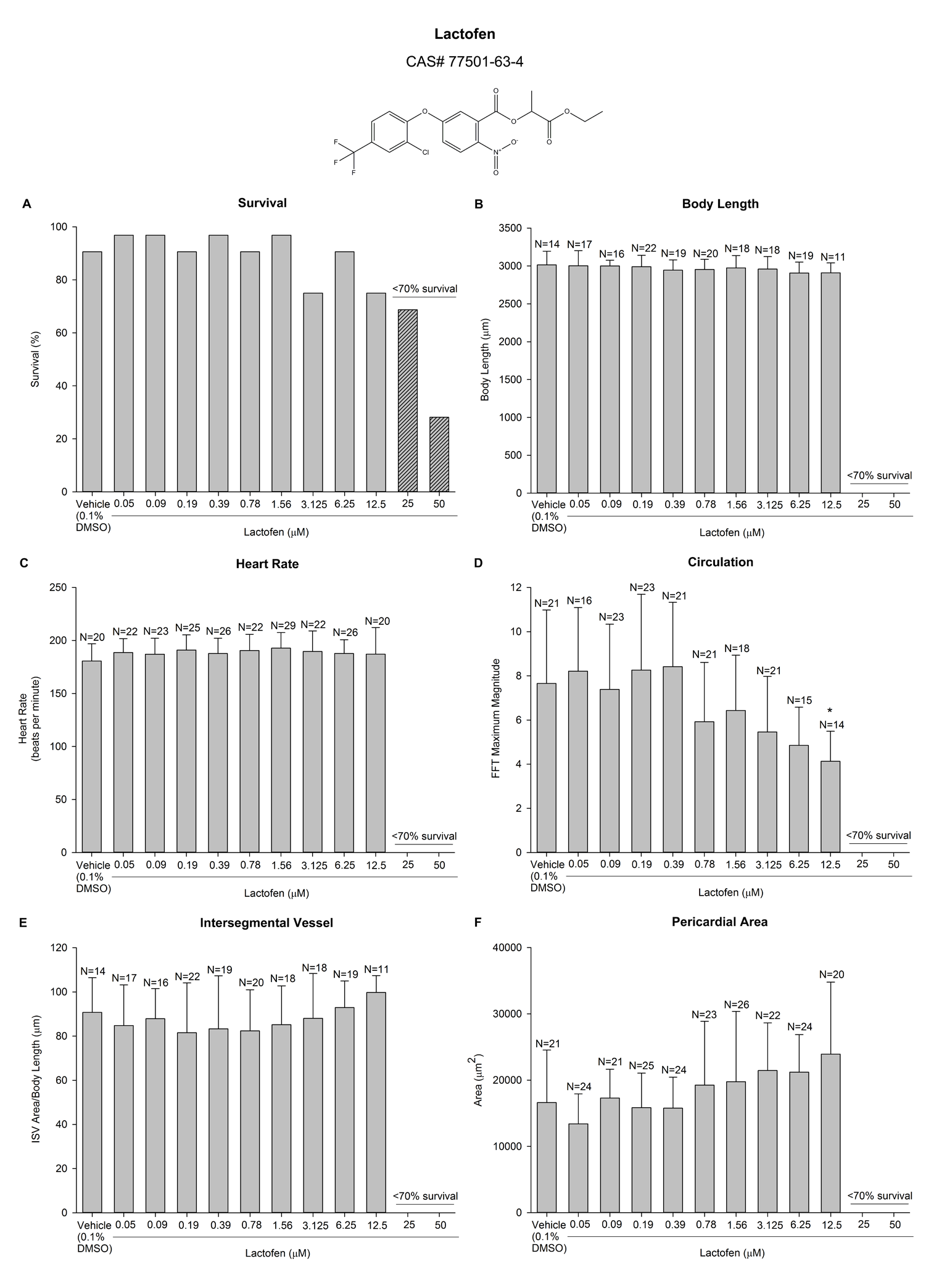

Supplement: Figure S18 — Lactofen did not have a concentration dependent effect on any endpoints. Based on decision criteria defined by Yozzo et al. [8], hashed bars represent concentrations that were not analyzed for potential effects on circulation, pericardial area, heart rate, or intersegmental vessel area. An asterisk denotes a significant difference from vehicle controls (p<0.05). N = final number of embryos analyzed per treatment. (TIF) [file pone.0104190.s018.tif]

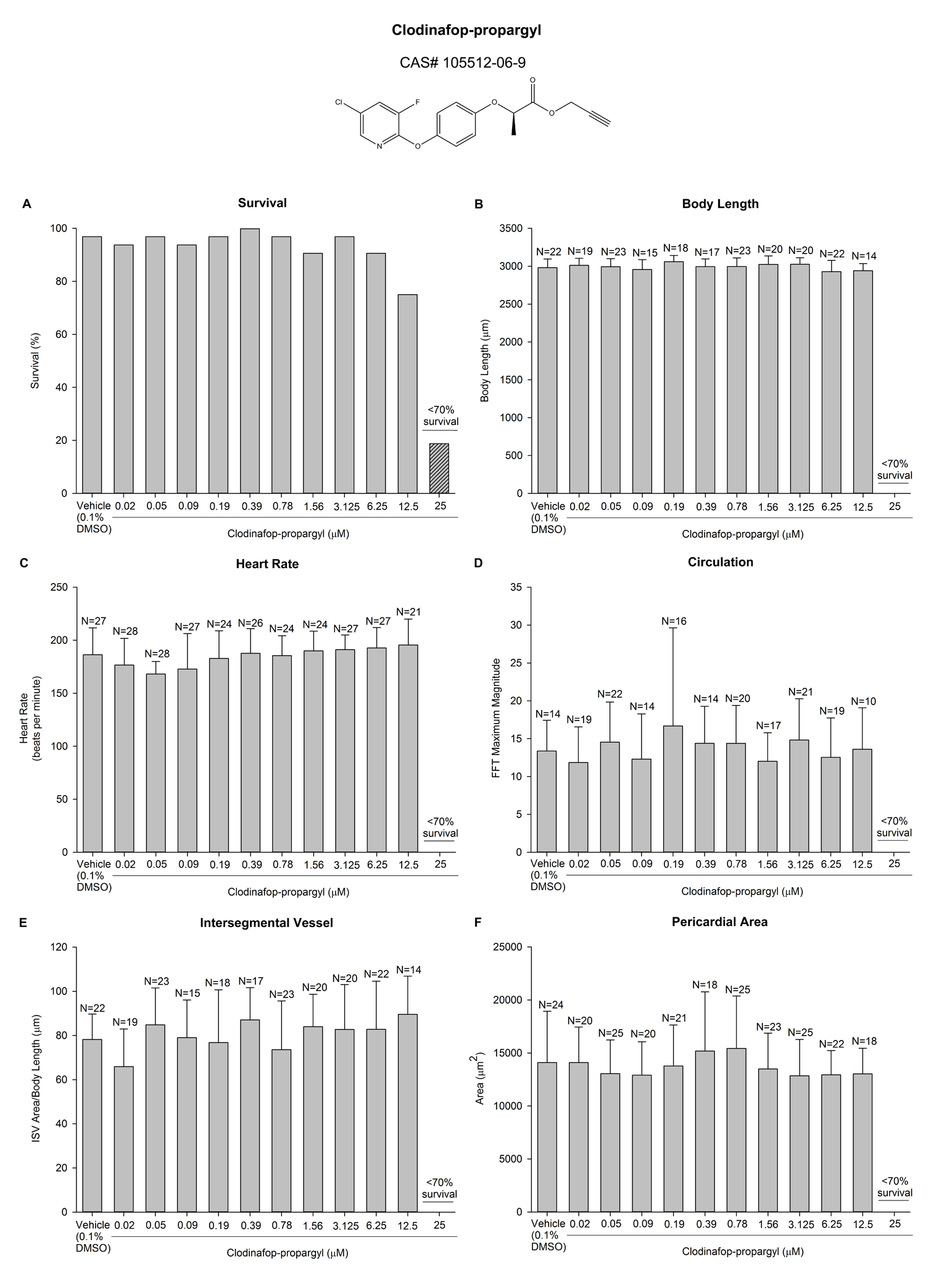

Supplement: Figure S19 — Clodinafop-propargyl did not have a concentration dependent effect on any endpoints. Based on decision criteria defined by Yozzo et al. [8], hashed bars represent concentrations that were not analyzed for potential effects on circulation, pericardial area, heart rate, or intersegmental vessel area. An asterisk denotes a significant difference from vehicle controls (p<0.05). N = final number of embryos analyzed per treatment. (TIF) [file pone.0104190.s019.tif]

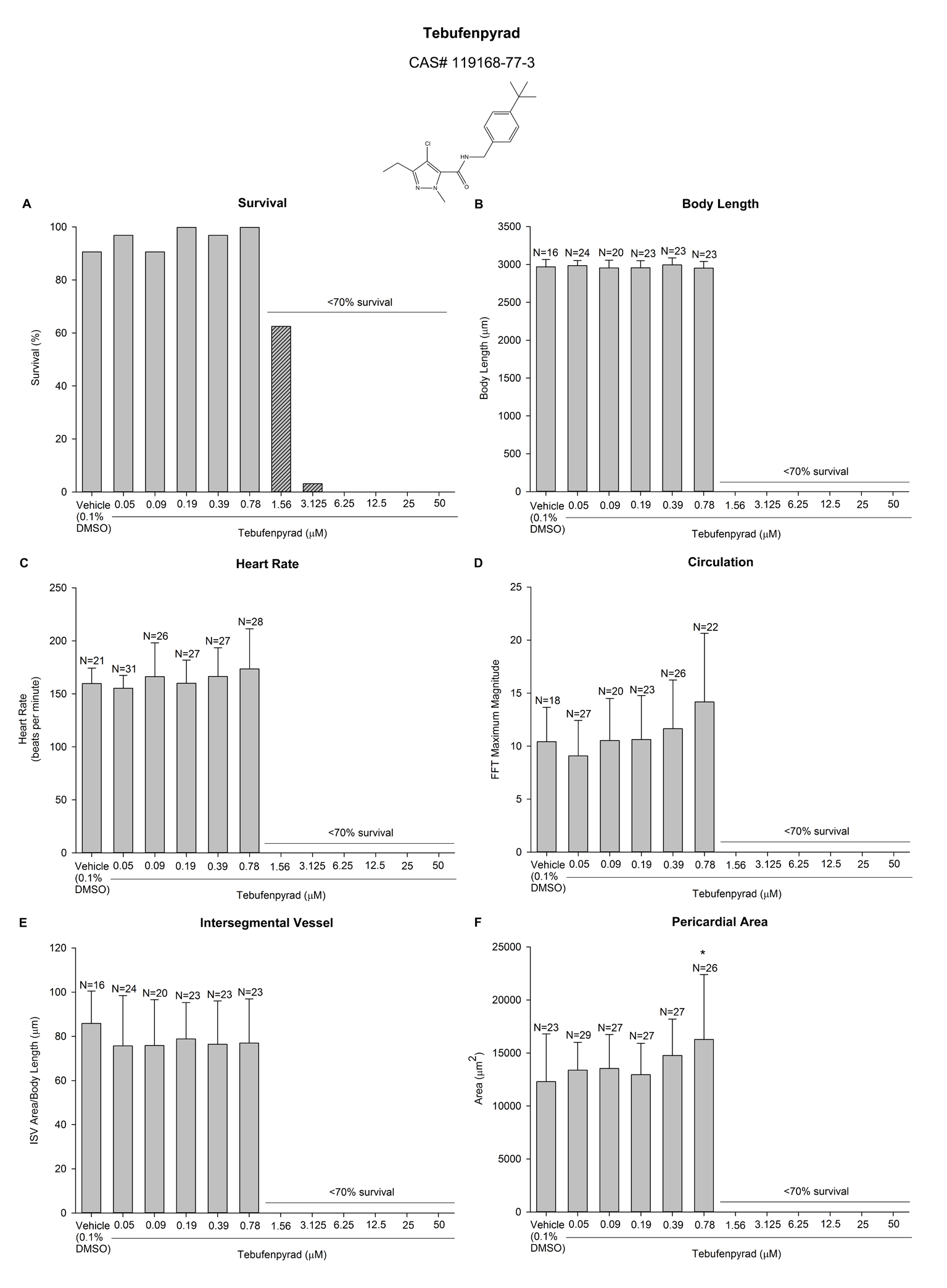

Supplement: Figure S20 — Tebufenpyrad did not have a concentration dependent effect on any endpoints. Based on decision criteria defined by Yozzo et al. [8], hashed bars represent concentrations that were not analyzed for potential effects on circulation, pericardial area, heart rate, or intersegmental vessel area. An asterisk denotes a significant difference from vehicle controls (p<0.05). N = final number of embryos analyzed per treatment. (TIF) [file pone.0104190.s020.tif]

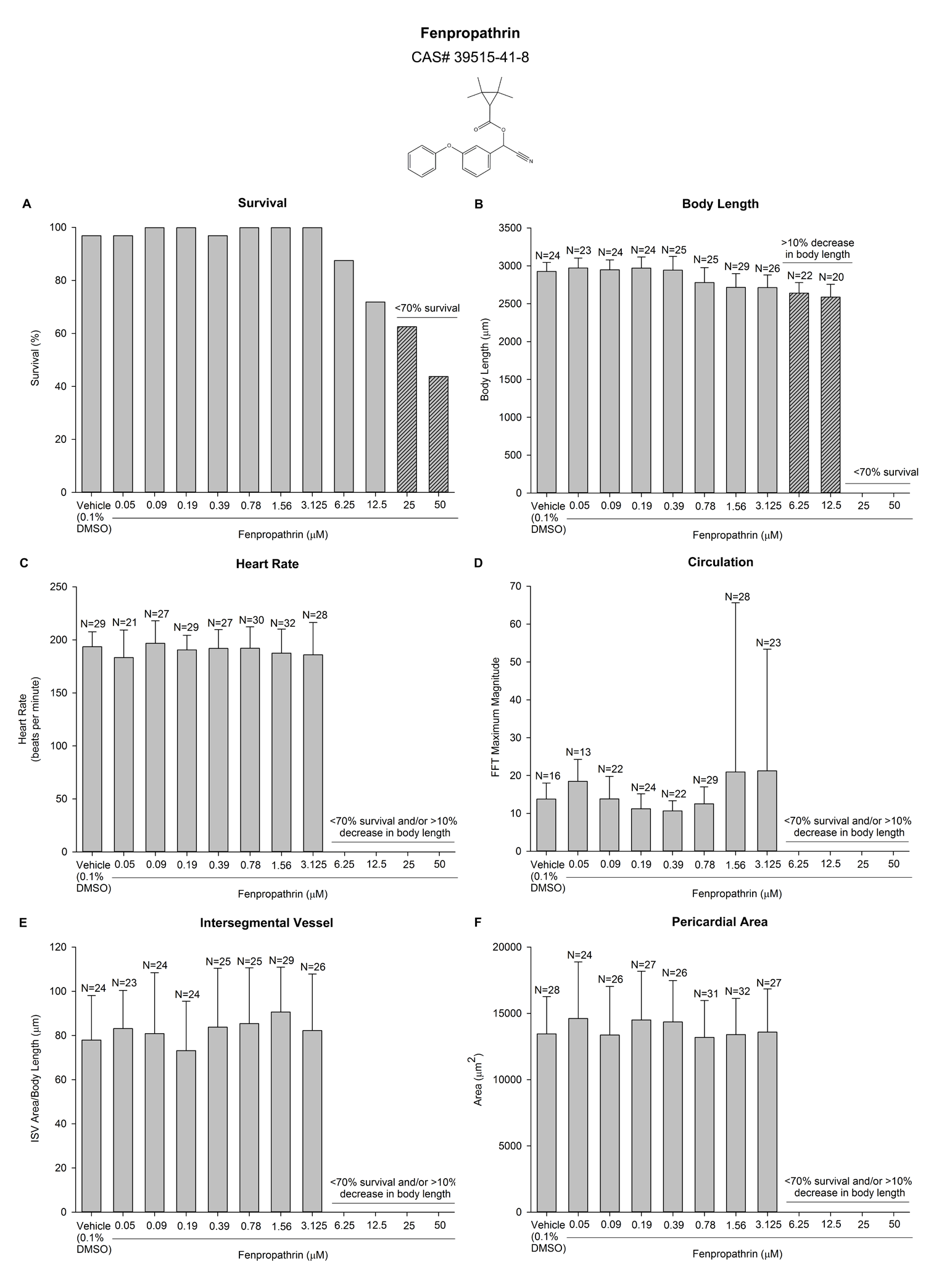

Supplement: Figure S21 — Fenpropathrin did not have a concentration dependent effect on any endpoints. Based on decision criteria defined by Yozzo et al. [8], hashed bars represent concentrations that were not analyzed for potential effects on circulation, pericardial area, heart rate, or intersegmental vessel area. An asterisk denotes a significant difference from vehicle controls (p<0.05). N = final number of embryos analyzed per treatment. (TIF) [file pone.0104190.s021.tif]

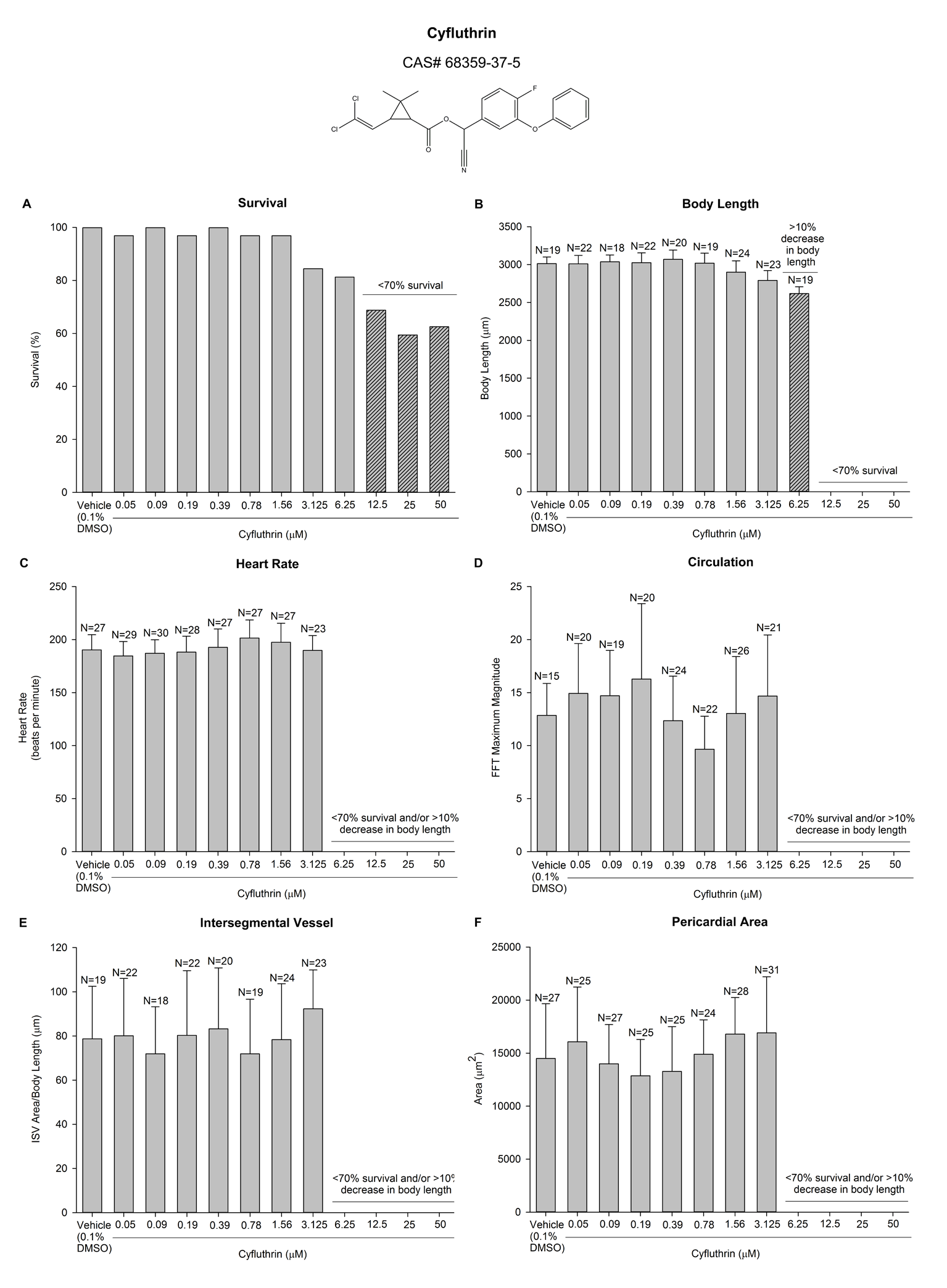

Supplement: Figure S22 — Cyfluthrin did not have a concentration dependent effect on any endpoints. Based on decision criteria defined by Yozzo et al. [8], hashed bars represent concentrations that were not analyzed for potential effects on circulation, pericardial area, heart rate, or intersegmental vessel area. An asterisk denotes a significant difference from vehicle controls (p<0.05). N = final number of embryos analyzed per treatment. (TIF) [file pone.0104190.s022.tif]

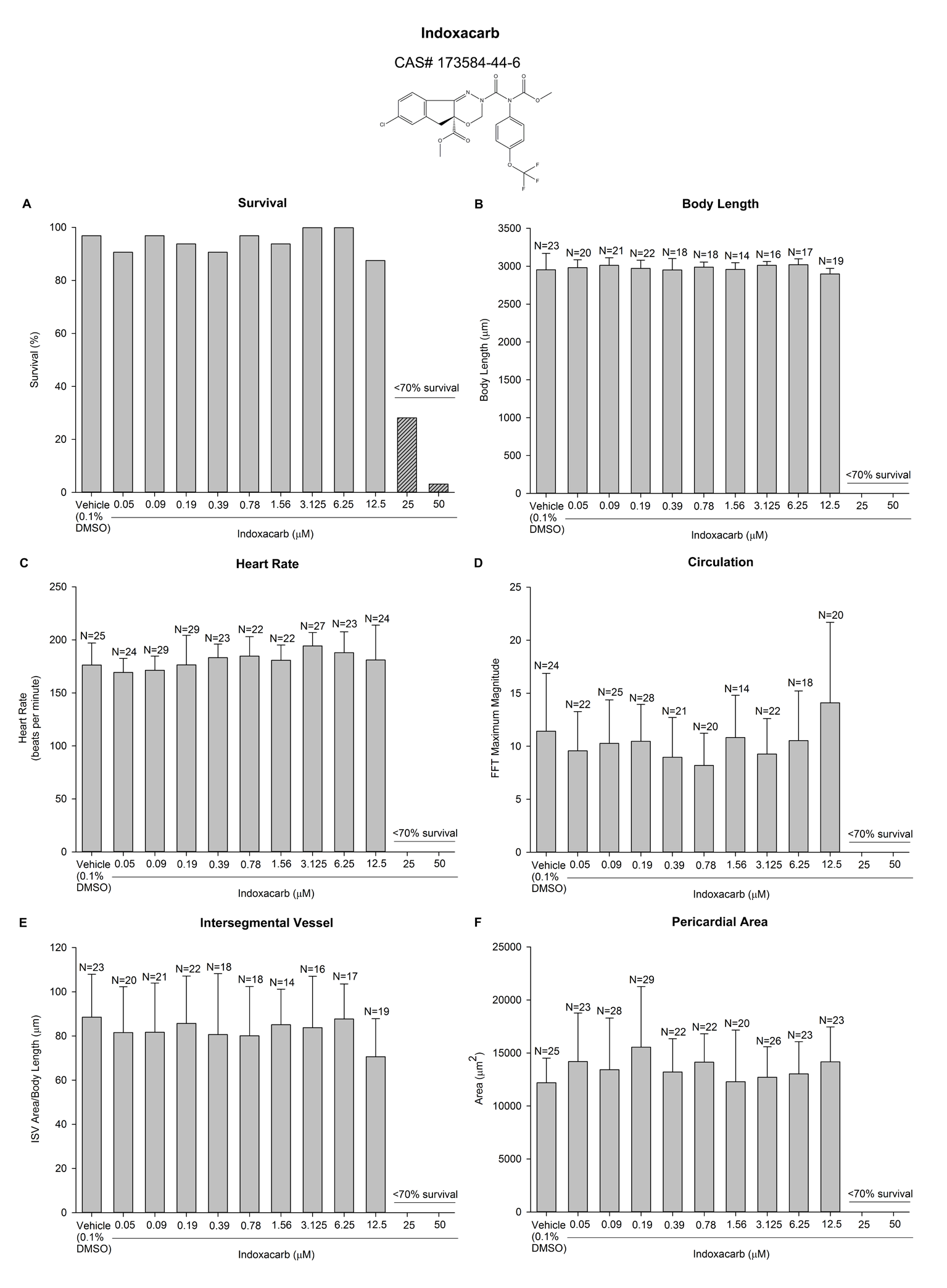

Supplement: Figure S23 — Indoxacarb did not have a concentration dependent effect on any endpoints. Based on decision criteria defined by Yozzo et al. [8], hashed bars represent concentrations that were not analyzed for potential effects on circulation, pericardial area, heart rate, or intersegmental vessel area. An asterisk denotes a significant difference from vehicle controls (p<0.05). N = final number of embryos analyzed per treatment. (TIF) [file pone.0104190.s023.tif]

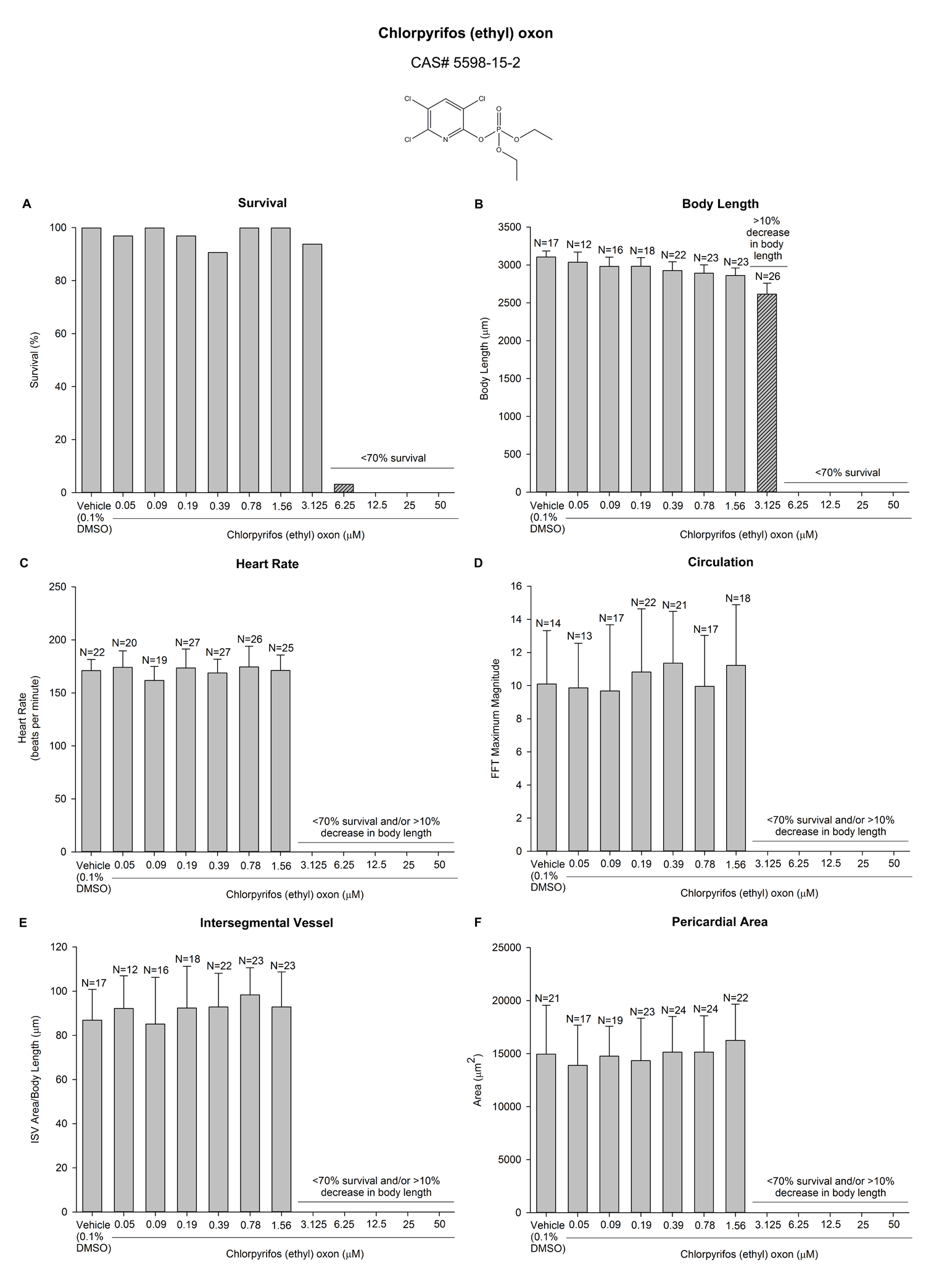

Supplement: Figure S24 — Chlorpyrifos (ethyl) oxon did not have a concentration dependent effect on any endpoints. Based on decision criteria defined by Yozzo et al. [8], hashed bars represent concentrations that were not analyzed for potential effects on circulation, pericardial area, heart rate, or intersegmental vessel area. An asterisk denotes a significant difference from vehicle controls (p<0.05). N = final number of embryos analyzed per treatment. (TIF) [file pone.0104190.s024.tif]

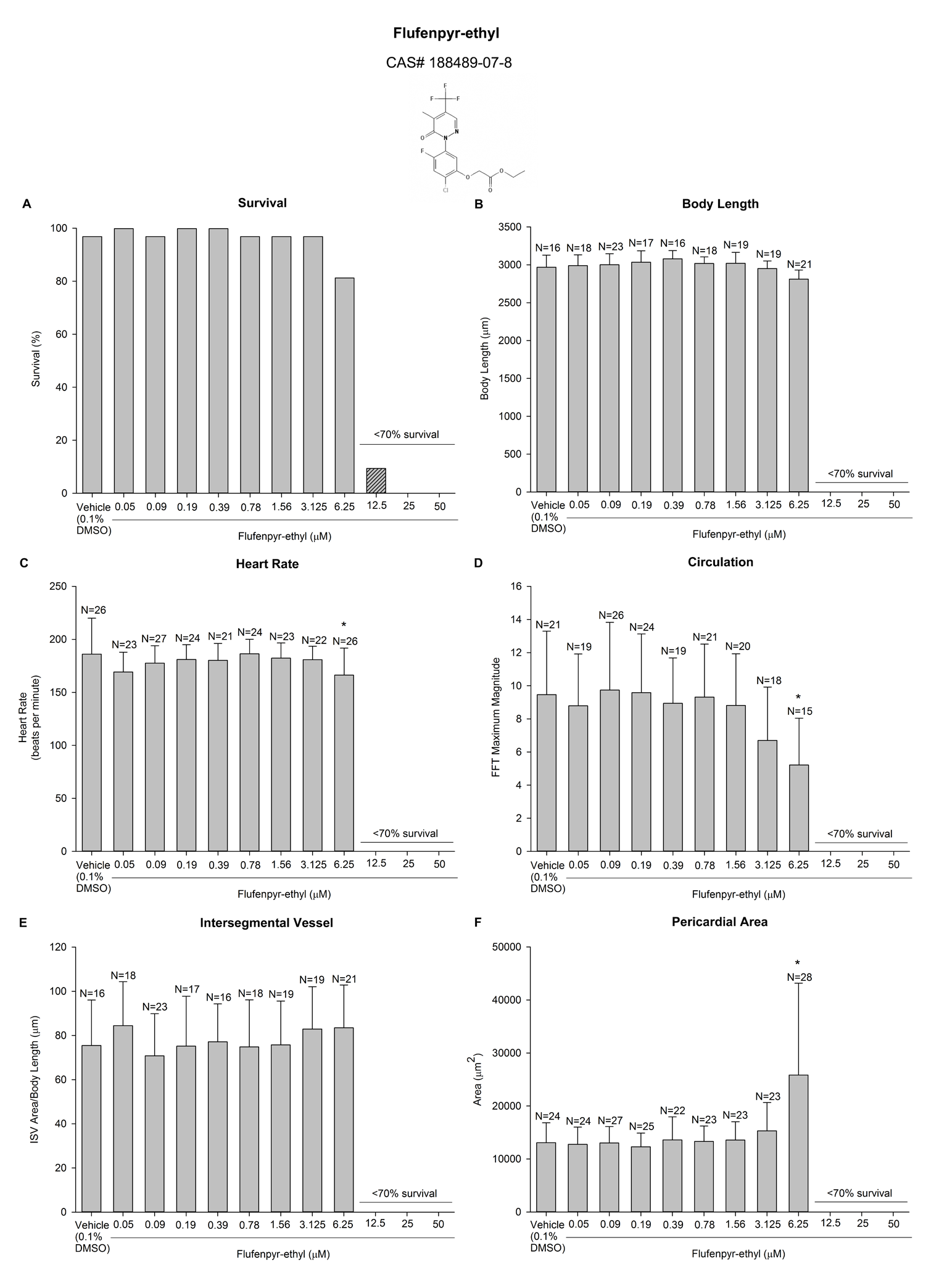

Supplement: Figure S25 — Flufenpyr-ethyl did not have a concentration dependent effect on any endpoints. Based on decision criteria defined by Yozzo et al. [8], hashed bars represent concentrations that were not analyzed for potential effects on circulation, pericardial area, heart rate, or intersegmental vessel area. An asterisk denotes a significant difference from vehicle controls (p<0.05). N = final number of embryos analyzed per treatment. (TIF) [file pone.0104190.s025.tif]

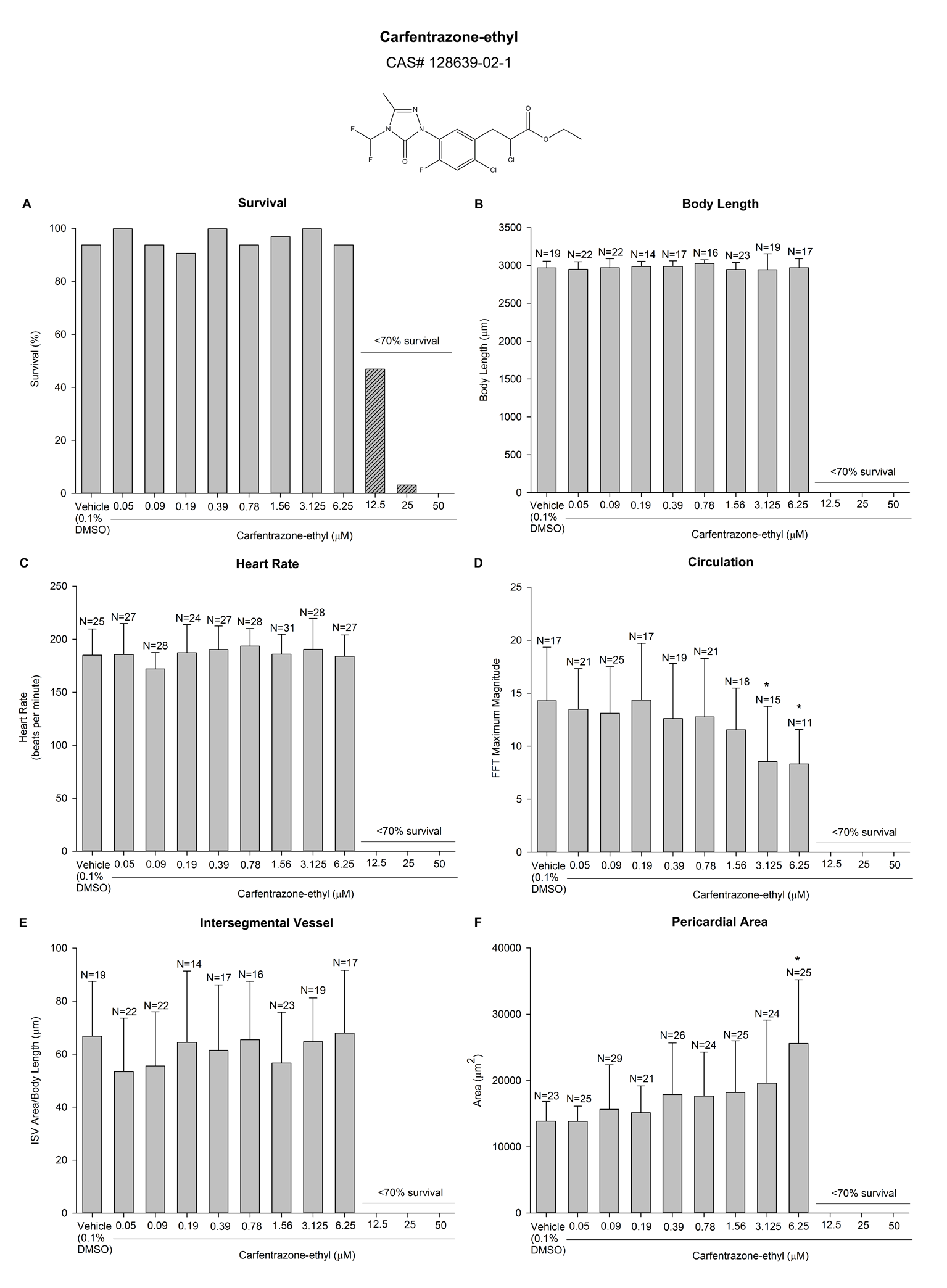

Supplement: Figure S26 — Carfentrazone-ethyl did not have a concentration dependent effect on any endpoints. Based on decision criteria defined by Yozzo et al. [8], hashed bars represent concentrations that were not analyzed for potential effects on circulation, pericardial area, heart rate, or intersegmental vessel area. An asterisk denotes a significant difference from vehicle controls (p<0.05). N = final number of embryos analyzed per treatment. (TIF) [file pone.0104190.s026.tif]

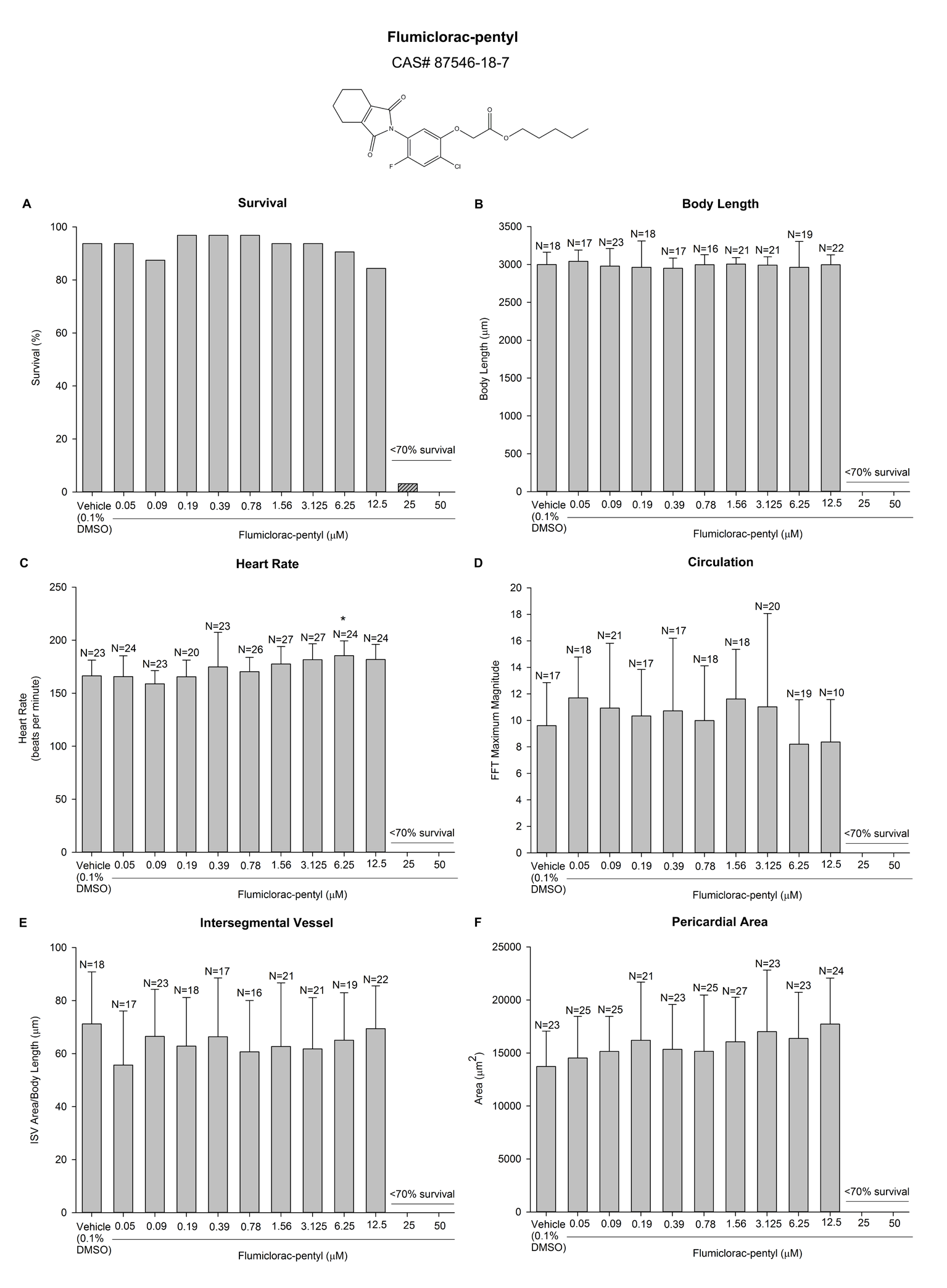

Supplement: Figure S27 — Flumiclorac-pentyl did not have a concentration dependent effect on any endpoints. Based on decision criteria defined by Yozzo et al. [8], hashed bars represent concentrations that were not analyzed for potential effects on circulation, pericardial area, heart rate, or intersegmental vessel area. An asterisk denotes a significant difference from vehicle controls (p<0.05). N = final number of embryos analyzed per treatment. (TIF) [file pone.0104190.s027.tif]

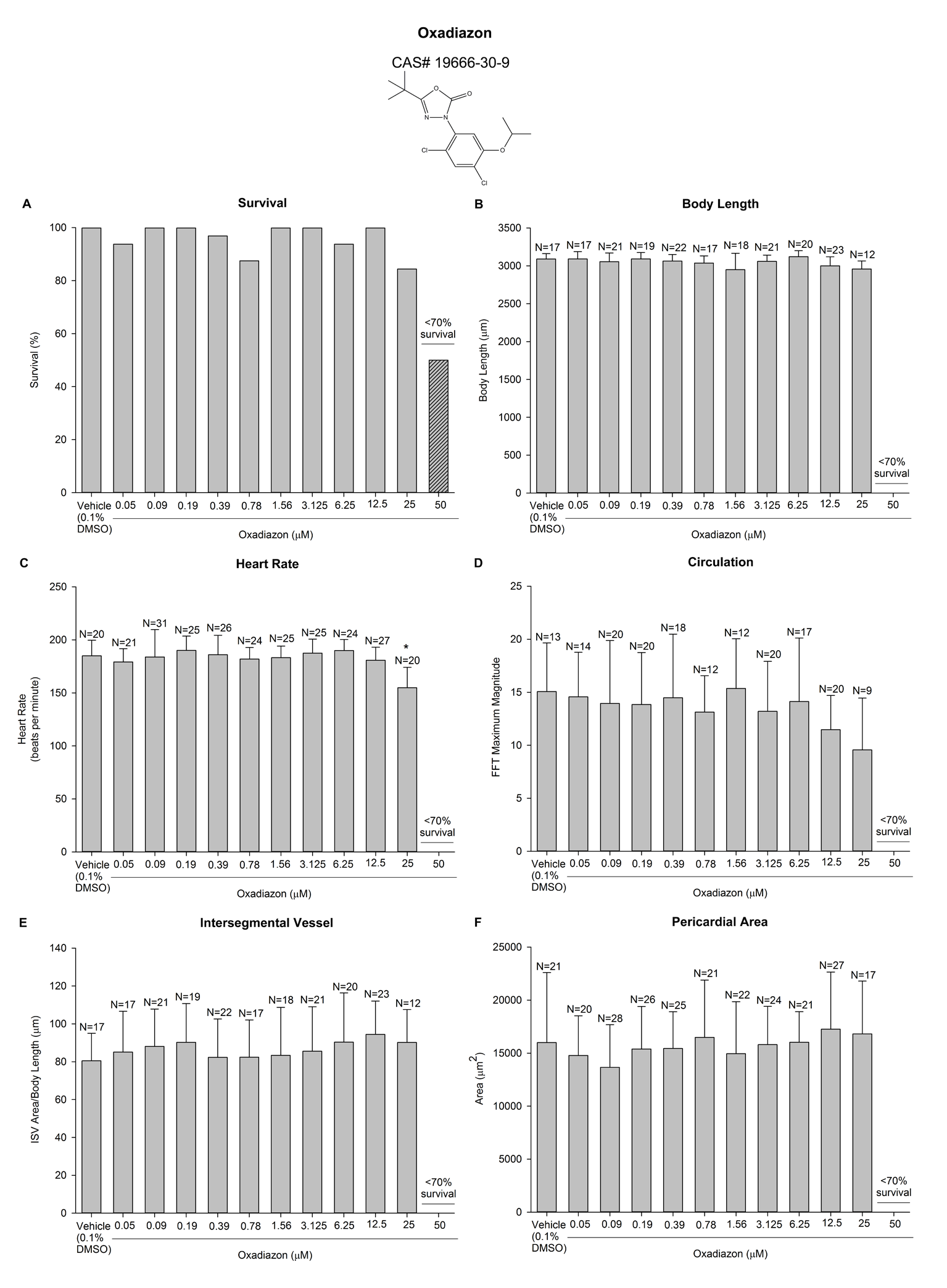

Supplement: Figure S28 — Oxadiazon did not have a concentration dependent effect on any endpoints. Based on decision criteria defined by Yozzo et al. [8], hashed bars represent concentrations that were not analyzed for potential effects on circulation, pericardial area, heart rate, or intersegmental vessel area. An asterisk denotes a significant difference from vehicle controls (p<0.05). N = final number of embryos analyzed per treatment. (TIF) [file pone.0104190.s028.tif]

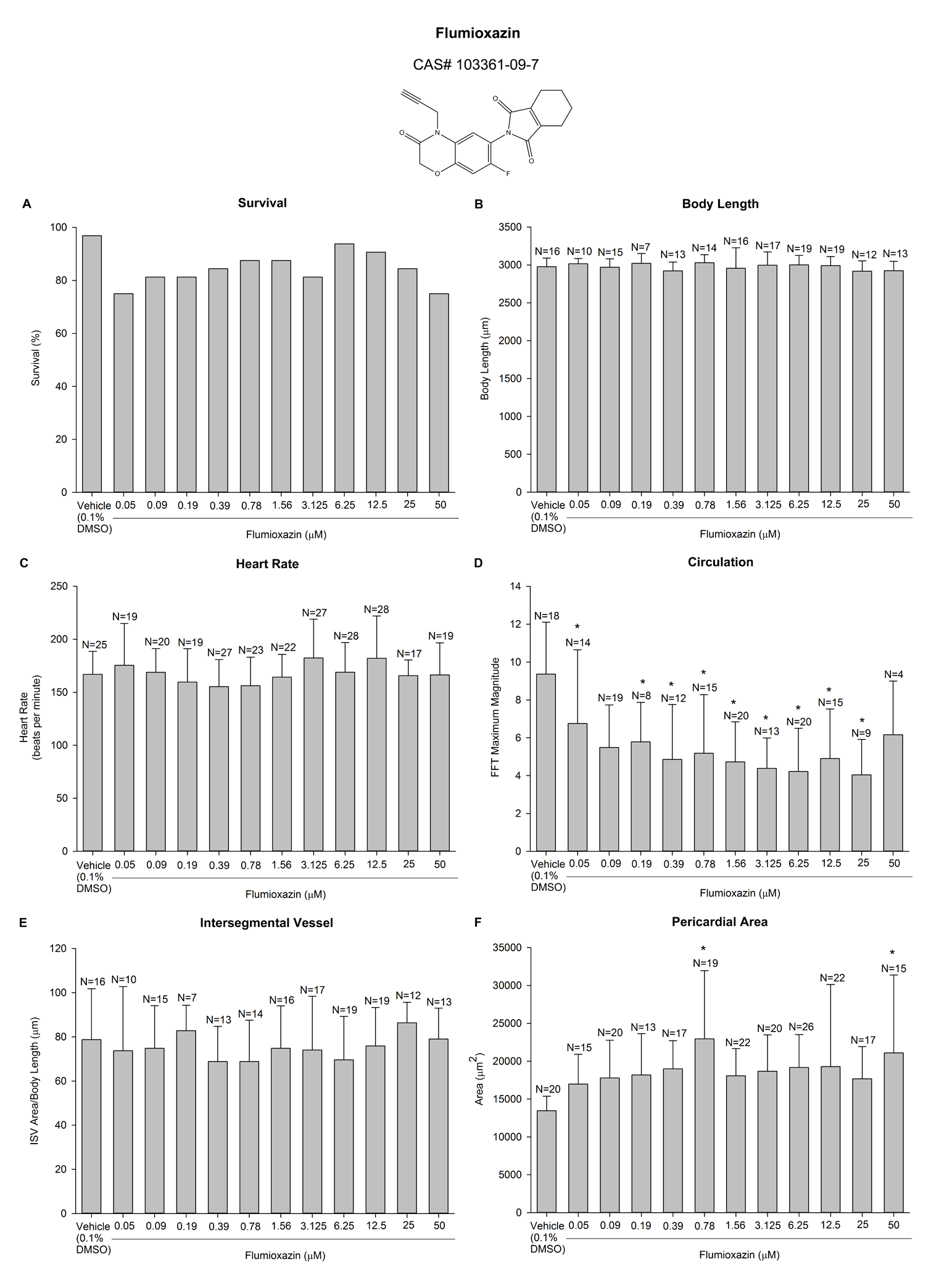

Supplement: Figure S29 — Flumioxazin did not have a concentration dependent effect on any endpoints. Based on decision criteria defined by Yozzo et al. [8], hashed bars represent concentrations that were not analyzed for potential effects on circulation, pericardial area, heart rate, or intersegmental vessel area. An asterisk denotes a significant difference from vehicle controls (p<0.05). N = final number of embryos analyzed per treatment. (TIF) [file pone.0104190.s029.tif]

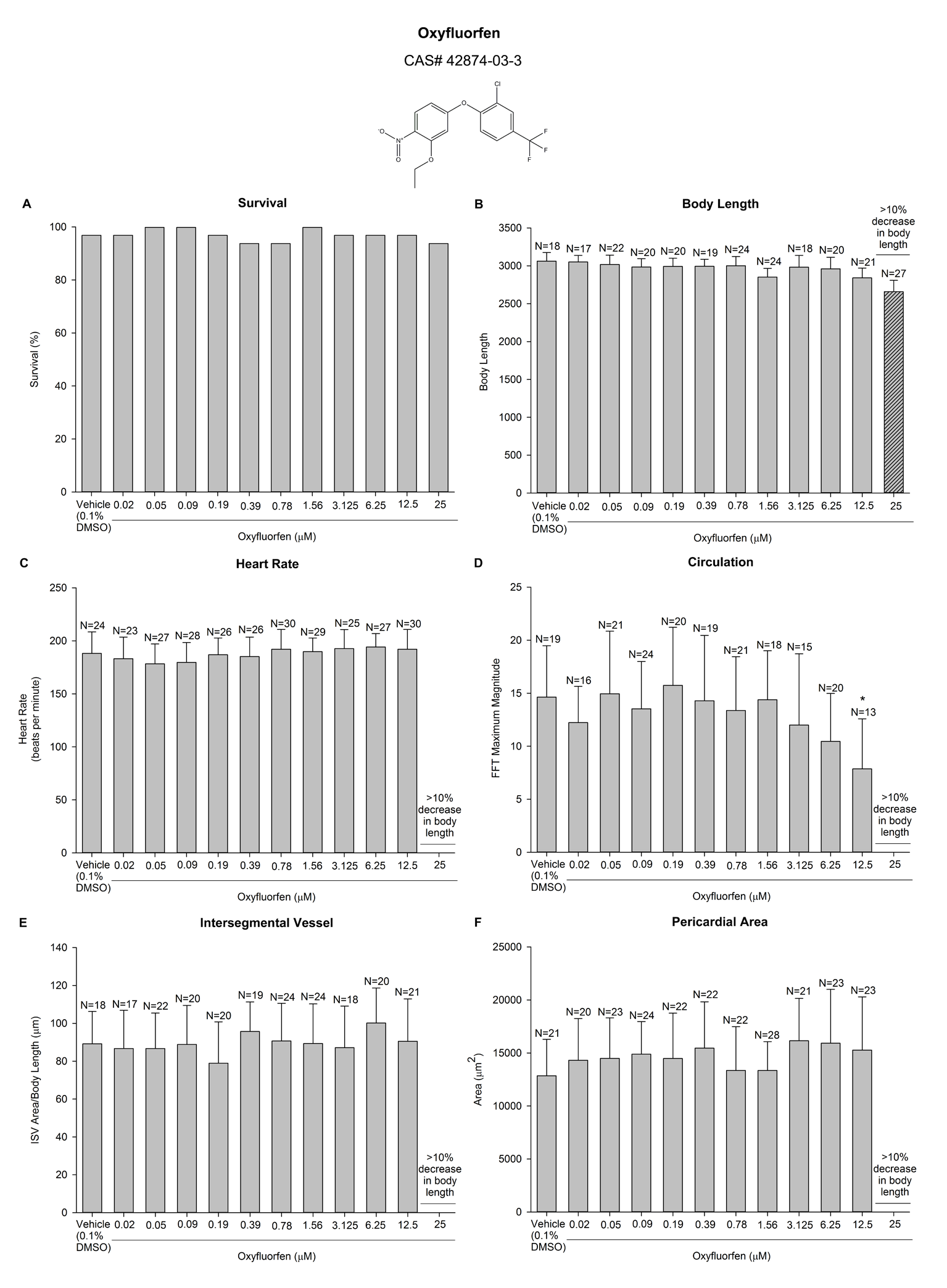

Supplement: Figure S30 — Oxyfluorfen did not have a concentration dependent effect on any endpoints. Based on decision criteria defined by Yozzo et al. [8], hashed bars represent concentrations that were not analyzed for potential effects on circulation, pericardial area, heart rate, or intersegmental vessel area. An asterisk denotes a significant difference from vehicle controls (p<0.05). N = final number of embryos analyzed per treatment. (TIF) [file pone.0104190.s030.tif]

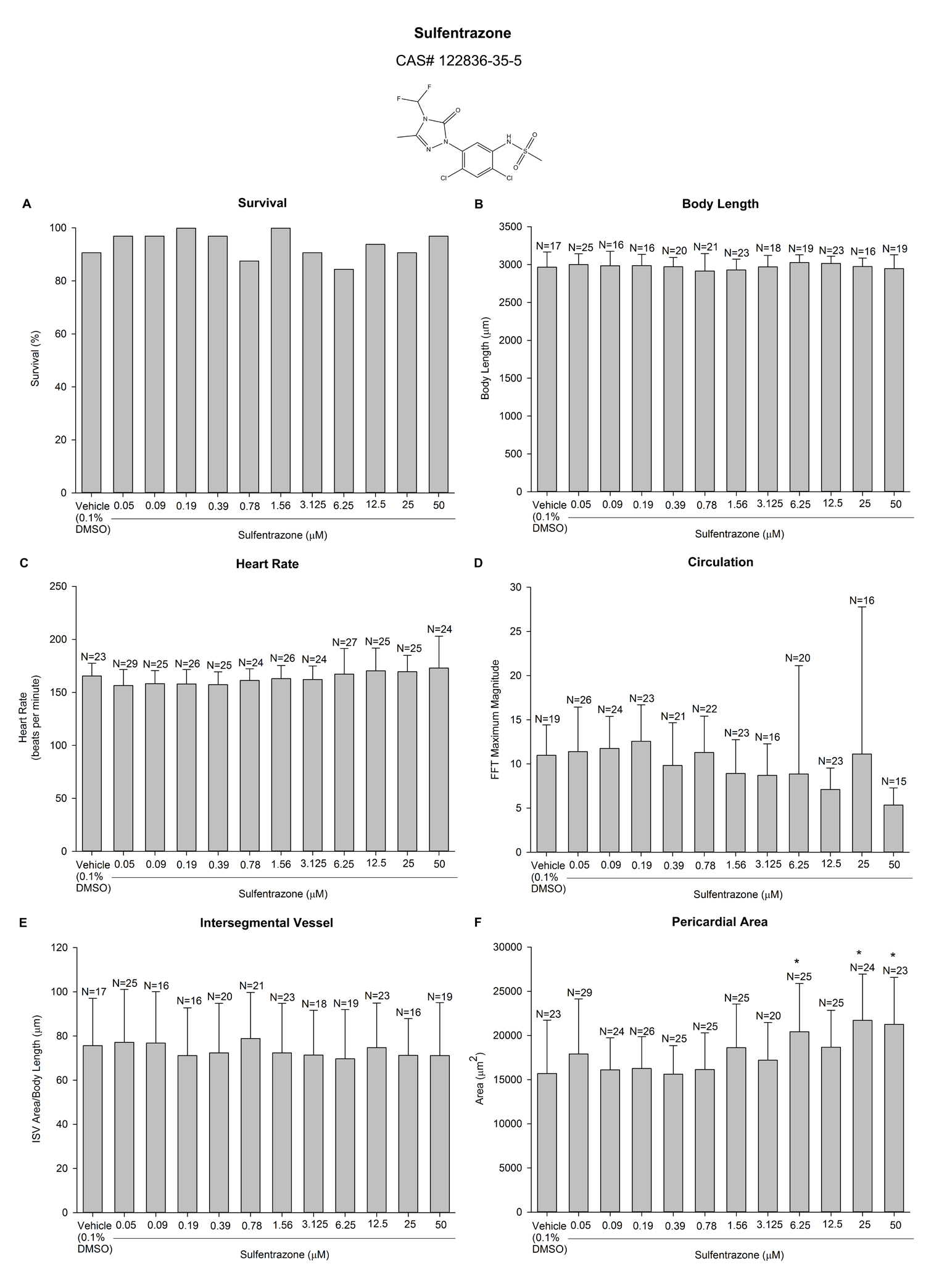

Supplement: Figure S31 — Sulfentrazone did not have a concentration dependent effect on any endpoints. Based on decision criteria defined by Yozzo et al. [8], hashed bars represent concentrations that were not analyzed for potential effects on circulation, pericardial area, heart rate, or intersegmental vessel area. An asterisk denotes a significant difference from vehicle controls (p<0.05). N = final number of embryos analyzed per treatment. (TIF) [file pone.0104190.s031.tif]

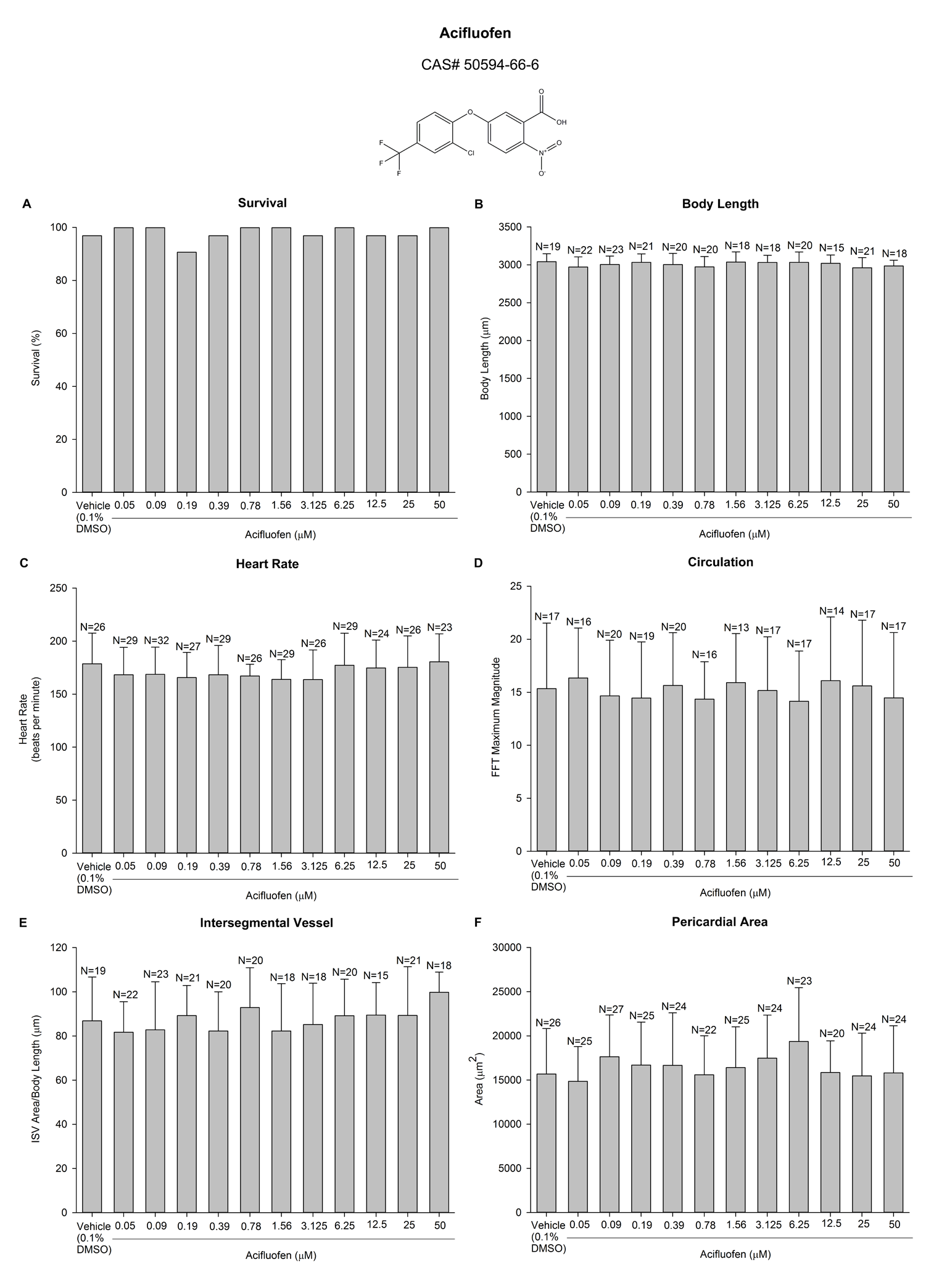

Supplement: Figure S32 — Acifluorfen did not have a concentration dependent effect on any endpoints. Based on decision criteria defined by Yozzo et al. [8], hashed bars represent concentrations that were not analyzed for potential effects on circulation, pericardial area, heart rate, or intersegmental vessel area. An asterisk denotes a significant difference from vehicle controls (p<0.05). N = final number of embryos analyzed per treatment. (TIF) [file pone.0104190.s032.tif]
