# Supplementary material for: High-Content Screening in Zebrafish Embryos Identifies Butafenacil as a Potent Inducer of Anemia
Source: PLoS One. 2014 Aug 4;9(8):e104190. doi: 10.1371/journal.pone.0104190 (PMC4121296; doi:10.1371/journal.pone.0104190)
Supplement: Table S1 — Chemical name, chemical formula, CAS registry number, vendor, and purity of all chemicals screened. (PDF) [file pone.0104190.s033.pdf]

Table S1. Chemical name, chemical formula, CAS registry number, vendor, and purity of all chemicals screened.

Chemical Name	Chemical Formula	CAS #	Vendor	Purity (%)
Rotenone	$C_{23}H_{22}O_6$	83-79-4	ChemService Inc.	97.8
Thiram	$C_6H_{12}N_2S_4$	137-26-8	ChemService Inc.	99.5
Butafenacil	$C_{20}H_{18}ClF_3N_2O_6$	134605-64-4	ChemService Inc.	99.3
Flumetralin	$C_{16}H_{12}ClF_4N_3O_4$	62924-70-3	ChemService Inc.	99.5
Fluthiacet-methyl	$C_{15}H_{15}ClFN_3O_3S_2$	117337-19-6	Sigma Aldrich	99.9
Abamectin	$C_{48}H_{72}O_{14}$ (B_{1a})<br>$C_{47}H_{70}O_{14}$ (B_{1b})	71751-41-2	ChemService Inc.	97.0
Propargite	$C_{19}H_{26}O_4S$	2312-35-8	ChemService Inc.	99.0
Pyraclostrobin	$C_{19}H_{18}ClN_3O_4$	175013-18-0	ChemService Inc.	99.5
(Z, E) – Fenproximate	$C_{24}H_{27}N_3O_4$	134098-61-6	ChemService Inc.	99.5
Tribufos	$C_{12}H_{27}OPS_3$	78-48-8	ChemService Inc.	98.1
Famoxadone	$C_{22}H_{18}N_2O_4$	131807-57-3	ChemService Inc.	98.5
Fluoxastrobin	$C_{21}H_{16}ClFN_4O_5$	361377-29-9	ChemService Inc.	99.5
Pyraflufen-ethyl	$C_{15}H_{13}Cl_2F_3N_2O_4$	129630-19-9	ChemService Inc.	99.2
Trifloxystrobin	$C_{20}H_{19}F_3N_2O_4$	141517-21-7	ChemService Inc.	99.3
Dazomet	$C_5H_{10}N_2S_2$	533-74-4	ChemService Inc.	98.0
Esfenvalerate	$C_{25}H_{22}ClNO_3$	66230-04-4	ChemService Inc.	99.5
Chlorothalonil	$C_8Cl_4N_2$	1897-45-6	ChemService Inc.	98.1
Lactofen	$C_{19}H_{15}ClF_3NO_7$	77501-63-4	ChemService Inc.	98.4
Clodinafop-propargyl	$C_{17}H_{13}ClFNO_4$	105512-06-9	ChemService Inc.	99.5
Tebufenpyrad	$C_{18}H_{24}ClN_3O$	119168-77-3	ChemService Inc.	99.5
Fenpropathrin	$C_{22}H_{23}NO_3$	64257-84-7	ChemService Inc.	99.4
Cyfluthrin	$C_{22}H_{18}Cl_2FNO_3$	68359-37-5	ChemService Inc.	98.0
Indoxacarb	$C_{22}H_{17}ClF_3N_3O_{\&}$	144171-61-9	ChemService Inc.	98.5
Chlorpyrifos (ethyl) oxon	$C_9H_{11}Cl_3NO_4P$	5598-15-2	ChemService Inc.	98.9
Flufenpyr-ethyl	$C_{16}H_{13}ClF_4N_2O_4$	188489-07-8	Laboratories Dr. Ehrenstorfer-Schafers	99.0
Carfentrazone-ethyl	$C_{15}H_{14}Cl_2F_3N_3O_3$	128639-02-1	ChemService Inc.	97.5
Flumiclorac-pentyl	$C_{21}H_{19}O_4NClF$	87546-18-7	ChemService Inc.	99.0
Oxadiazon	$C_{15}H_{18}Cl_2N_2O_3$	19666-30-9	ChemService Inc.	99.5
Flumioxazin	$C_{19}H_{15}FN_2O_4$	103361-09-7	ChemService Inc.	98.5
Oxyfluorfen	$C_{15}H_{11}ClF_3NO_4$	42874-03-3	ChemService Inc.	98.5
Sulfentrazone	$C_{11}H_{10}Cl_2F_2N_4O_3S$	122836-35-5	ChemService Inc.	99.4
Acifluorfen	$C_{14}H_7ClF_3NO_5$	50594-66-6	ChemService Inc.	99.5
